# Infrapatellar Fat Pad Glucocorticoid Injection in Knee Osteoarthritis: A Randomized Clinical Trial

**DOI:** 10.1001/jamanetworkopen.2025.49938

**Published:** 2026-01-02

**Authors:** Yan Zhang, Guangfeng Ruan, Tao Fan, Peng Zheng, Zhaohua Zhu, Juanjuan He, Xiaomei Wei, Wenjie Hu, Sili Huang, Qing Chang, Peichun Gao, Haowei Chen, Xiaoni Zhou, Xuelian Liu, Su’an Tang, Li Jiang, Changhai Ding

**Affiliations:** 1 Clinical Research Center, Zhujiang Hospital, Southern Medical University, Guangzhou, Guangdong, China; 2 Institute of Exercise and Rehabilitation Science, Zhujiang Hospital, Southern Medical University, Guangzhou, Guangdong, China; 3 Clinical Research Center, Guangzhou First People’s Hospital, School of Medicine, South China University of Technology, Guangzhou, China; 4 Department of Rehabilitation Medicine, Zhujiang Hospital, Southern Medical University, Guangzhou, Guangdong, China; 5 Department of Rehabilitation Medicine, The Third Affiliated Hospital of Sun Yat-Sen University, Guangzhou, Guangdong, China; 6 Department of Rehabilitation Medicine, The Sixth Affiliated Hospital of Sun Yat-Sen University, Guangzhou, Guangdong, China; 7 Department of Rehabilitation Medicine, The Third Affiliated Hospital of Southern Medical University, Guangzhou, Guangdong, China; 8 Department of Rehabilitation Medicine, Beihai People’s Hospital, Beihai, Guangxi, China; 9 Department of Spinal Surgery, Orthopedic Medical Center, Zhujiang Hospital, Southern Medical University, Guangzhou, Guangdong, China

## Abstract

**Importance:**

Intra-articular glucocorticoid injections are widely used to alleviate knee osteoarthritis (OA) pain, but evidence suggests these injections may cause cartilage loss. The infrapatellar fat pad (IPFP) and synovium are important sources of inflammation in knee OA; injecting glucocorticoid into the IPFP may not only provide anti-inflammatory effects but also reduce cartilage deterioration in patients with inflammatory knee OA.

**Objective:**

To evaluate the effect and safety of glucocorticoid injections into the IPFP in individuals with inflammatory knee OA.

**Design, Setting, and Participants:**

This randomized, double-blind, placebo-controlled trial included patients aged 45 years and older with inflammatory knee OA at 4 centers in China. Patients were enrolled from April 2022 to June 2023.

**Intervention:**

Patients were randomly assigned to the treatment group (n = 30) or placebo group (n = 30). Each group received either glucocorticoid or saline injections into the IPFP with hyaluronic acid as background treatment under ultrasonographic guidance.

**Main Outcomes and Measures:**

The primary outcomes were changes in knee pain on a visual analog scale (VAS) and effusion synovitis volume measured by magnetic resonance imaging (MRI). Secondary outcomes included changes in the total score of the Western Ontario and McMaster Universities Osteoarthritis Index (WOMAC), MRI-detected Hoffa synovitis score, quality of life assessed using the 4-dimensional Assessment of Quality of Life, pain medication use, IPFP volume, and the incidence of adverse reactions. Outcomes were assessed over 12 weeks.

**Results:**

All 60 participants (mean [SD] age, 65 [11] years; 38 [63%] women) completed the study. The treatment group compared with the placebo group did not have a statistically significant reduction in VAS pain (−39.3 mm vs −31.4 mm; between-group difference, −7.9 mm; 95% CI, −19.7 to 4.0 mm). There was no significant between-group difference in effusion volume reduction (−4.9 mL vs −5.4 mL; between-group difference, 0.5 mL; 95% CI, −1.9 to 2.9 mL). In post hoc analyses, the treatment group had significantly greater reduction in the WOMAC pain score (−113.0 points vs −66.8 points; between group difference, −46.2 points; 95% CI, −90.0 to −2.4 points; *P* = .04) and cartilage defect (−0.1 vs 0.4; between-group difference, −0.5; 95% CI, −1.0 to −0.1; *P* = .03). Both groups had 1 participant who experienced 1 adverse reaction.

**Conclusions and Relevance:**

In this randomized clinical trial, glucocorticoid injections into the IPFP did not effectively alleviate knee pain or reduce effusion synovitis volume in inflammatory knee OA. Further investigation is needed to determine the efficacy of this treatment approach.

**Trial Registration:**

ClinicalTrials.gov Identifier: NCT05291650

## Key Points

**Question:**

Is the administration of glucocorticoid injections into the infrapatellar fat pad (IPFP) effective and safe for individuals with inflammatory knee osteoarthritis (OA)?

**Findings:**

This randomized clinical trial of 60 patients found that the treatment group, compared with the placebo group, did not experience a statistically significant reduction in visual analog scale pain scores (−39.3 mm vs −31.4 mm). Both groups showed a reduction in effusion volume, with no significant between-group difference.

**Meaning:**

The negative finding of this intra-IPFP glucocorticoid injections trial suggests further work is needed to identify effective interventions in patients with inflammatory knee OA.

## Teaser

This randomized clinical trial evaluates whether glucocorticoid injections in the infrapatellar fat pads reduce pain and inflammation among adults aged 45 years and older with inflammatory knee osteoarthritis in China.

## Introduction

Osteoarthritis (OA) is a highly prevalent joint disease that affects approximately 595 million people worldwide, and the knee is the most commonly affected joint.^1^ Symptomatic knee OA often leads to physical disability, reduced quality of life, and increased mortality in older adults.^2^ Despite the increasing personal, economic, and societal burden of knee OA due to the aging population and obesity epidemic, curative drugs for knee OA remain lacking.^2^

Accumulating evidence suggests that knee OA encompasses diverse phenotypes characterized by distinct pathological changes, and one notable phenotype is inflammatory knee OA.^3^ The infrapatellar fat pad (IPFP) and synovium, structurally interconnected, are considered an integrated tissue unit and important sources of inflammation in knee OA.^4^ IPFP, an adipose tissue, is below the patella and located close to the synovial layers and is abundant with adipocytes and immune cells.^5^ Abnormal IPFP can release a variety of inflammatory products that result in changes of the cartilage, synovium, and subchondral bone, ultimately accelerating OA progression.^5^ Signal alterations in IPFP detected by magnetic resonance imaging (MRI) are referred to as Hoffa synovitis.^6^ Abundant clinical evidence showed that Hoffa synovitis was associated with structural and symptomatic abnormalities in the knee.^7,8,9^ Beyond these clinical associations, basic research has shown that IPFP alterations may play a critical role in knee OA.^10,11,12,13,14^ Joint effusion shown on noncontrast enhanced MRI reflects a composite of effusion and synovial thickening, known as effusion synovitis.^15^ Effusion synovitis is prevalent in more than 46% of patients with knee OA and is significantly associated with increased knee pain.^16^ Also, it is associated with structural issues, such as cartilage defects, bone marrow lesions, and cartilage volume loss, and with an increased likelihood of needing joint replacement surgery.^17^ Therefore, anti-inflammatory therapies for knee OA patients with signal alterations in the IPFP and effusion synovitis may slow deleterious structural changes and improve clinical outcomes in inflammatory knee OA.

In knee OA management, intra-articular glucocorticoid injections are recommended to alleviate inflammation and pain.^18,19^ However, they are not considered a first-line therapy and are generally reserved for patients when other pharmacological treatments are ineffective or unsuitable or to support therapeutic exercise.^20^ A systematic review found that intra-articular glucocorticoid injections were effective in reducing pain compared with placebo in the short term (follow-up ≤6 weeks; standardized mean difference [SMD], −0.61).^21^ However, the magnitude of pain relief decreased over time (1-2 weeks, moderate; 4-6 weeks, small to moderate; 13 weeks, small; 26 weeks, no evidence of benefit).^22^ More importantly, a randomized clinical trial (RCT) revealed that such injections may induce cartilage loss.^23^ This could be attributed to glucocorticoids triggering matrix catabolism and chondrocyte senescence.^24,25^ Due to the short duration of symptom relief and concerns about potential cartilage damage, the clinical benefits of intra-articular glucocorticoid injections for knee OA remain controversial. Injecting glucocorticoid into IPFP may not only provide anti-inflammatory effects but also minimize cartilage deterioration in patients with inflammatory knee OA. Therefore, we conducted a trial to evaluate whether glucocorticoid injections into the IPFP effectively alleviate knee pain and reduce effusion synovitis volume in individuals with inflammatory knee OA characterized by effusion synovitis and Hoffa synovitis.

## Methods

### Trial Design

Glucocorticoid Injections Into the Infrapatellar Fat Pad in Patients With Knee Osteoarthritis (GLITTERS) was a 12-week multicenter, randomized, double-blind, placebo-controlled trial. Recruitment of participants took place from April 2022 to June 2023 in 4 centers in China. Ethics approval was obtained from the Zhujiang Hospital Ethics Committee, Guangzhou, China; The Sixth Affiliated Hospital of Sun Yat-sen University Ethics Committee; The Third Affiliated Hospital of Sun Yat-sen University Ethics Committee; and the Beihai People’s Hospital Ethics. Written informed consent was obtained from each participant. All procedures involving human participants will be performed in accordance with the 1964 Helsinki Declaration and the regulations for clinical trials in China. Details of the trial design have been published previously,^26^ and the full trial protocol is available in Supplement 1. This report follows the Consolidated Standards of Reporting Trials (CONSORT) 2025 statement.

### Setting and Participants

Between April 22, 2022, and June 13, 2023, 141 Chinese participants were screened. Following the screening visit, participants were assessed at weeks 0 (baseline), 4, 8, and 12. For the 4-week and 8-week follow-ups, participants could complete the visit at home via questionnaires and a remote interview. MRI scans were performed only at baseline and at 12 weeks. Detailed information is available in the data collection schedule in the protocol (Supplement 1).^26^

The inclusion and exclusion criteria were outlined in the published protocol.^26^ In summary, individuals were considered eligible if they were aged 45 years or older, met the symptomatic knee OA criteria according to the American College of Rheumatology standards,^27^ and experienced knee pain persisting for more than 6 months, with pain assessed by a visual analog scale (VAS; range, 100 mm) and a score of 40 mm or greater in the last week. Additionally, participants were required to have an MRI-assessed Hoffa synovitis score (using the MRI Osteoarthritis Knee Score [MOAKS method]^28,29^) of 1 or greater and an effusion synovitis score (MOAKS method)^28^ of 1 or greater, with a total score of 3 or greater. Exclusion criteria encompassed the following conditions: allergy to glucocorticoids; knee joint injection of glucocorticoid or hyaluronic acid within the past 6 months; severe trauma or arthroscopy in the knee within the past 6 months; planned hip or knee surgery in the next 6 months; contraindication to having MRI; presence of other arthritis, other conditions causing more pain than their knee OA, malignant tumors or other life-threatening diseases, infection, diabetes, coagulopathy, osteonecrosis, or gastric or duodenal ulcer within the past 12 months; current use of oral corticosteroids, nonsteroidal anti-inflammatory drugs, or immunosuppressive medication; pregnancy or lactating female; and the use of any investigational drugs or devices in the past 30 days. In cases where both of the participants’ knees met the eligibility criteria, the knee with more severe VAS pain was selected as the study knee.

### Randomization and Blinding

Randomization was stratified by center and performed using computer-generated blocks of 4 in a 1:1 ratio. Drugs were packaged in identical opaque boxes labeled by participant identification according to the assignment list, thereby ensuring allocation concealment. Participants and researchers, including assessors and statisticians, were blinded until data analysis. Injecting therapists were unblinded but not involved in other aspects of the trial.

### Interventions

Under ultrasonography guidance at baseline, both the glucocorticoid group (receiving Compound Betamethasone Injectable, Diprosone [Schering-Plough Labo N.V.]) and the placebo group (receiving saline) received 1 IPFP injection. Each 1 mL of the glucocorticoid contained 5 mg of betamethasone dipropionate and 2 mg of betamethasone sodium phosphate (calculated as betamethasone). The IPFP injection included either 1 mL of glucocorticoid with 0.5 mL of saline and 0.5 mL of lidocaine (Lidocaine Hydrochloride lnjection, concentration: 2% [Shanghai Chaohui Pharmaceutical Co, Ltd]) for the treatment group or 1.5 mL of saline and 0.5 mL of lidocaine for the placebo group. As detailed in the protocol,^26^ the drug was injected at 2 sites at the bottom of the IPFP near the synovium, where synovial hyperplasia is evident, with 1 mL administered at each site. To facilitate patient recruitment and obtain ethical approval, an intra-articular injection of 2.5 mL hyaluronic acid (sodium hyaluronate injection, ARTZ Dispo [Seikagaku Corp]) was administered as concomitant treatment in both groups. Each vial (2.5 mL) contains sodium hyaluronate 25 mg. The doses of glucocorticoid and hyaluronic acid used in this study were based on the standard clinical doses commonly used for intra-articular injection in the management of knee OA.

### Outcomes

The primary outcomes were change in knee pain at the 12-week follow-up as well as effusion synovitis volume at 12-week follow-up. Knee pain was assessed using the VAS^30^ at baseline and the 3 follow-ups. Effusion synovitis volume was evaluated on MRI at baseline and 12-week follow-up by calculating the sum of the volumes of effusion synovitis in the suprapatellar pouch, central portion, posterior femoral recess, and subpopliteal recess using OsiriX software version 12.1.0.^17^ The volumes of individual joint subregions were assessed by selecting each region of interest based on the intra-articular fluid-equivalent signal on a section-by-section basis. The final 3-dimensional volume rendering was generated using OsiriX MD imaging software.

The secondary outcomes involved evaluating changes in Western Ontario and McMaster Universities Osteoarthritis Index (WOMAC), quality of life, and pain medication usage at 12-week follow-up. Additionally, the Hoffa synovitis score (MOAKS) and infrapatellar fat pad volume on MRI at 12-week follow-up were included. Furthermore, adverse reactions were recorded at each follow-up, and the number of participants experiencing at least 1 adverse reaction was reported as a secondary outcome. Post hoc outcomes included changes in the WOMAC pain, stiffness, and function scores, along with protocol-specified other measures: depression (9-item Patient Health Questionnaire [PHQ-9]^31^) and MRI-assessed scores for effusion synovitis (MOAKS^28^), bone marrow lesion size,^26^ and cartilage defects.^26^ For the MRI assessments, detailed MRI sequences and parameters at the 4 study sites were provided in eTable 1 in Supplement 2. eTable 2 in Supplement 2 presented the intrareader and interreader reliability as well as the smallest detectable change for the MRI assessments. Further details on outcome assessments were provided in the eMethods in Supplement 2.

### Sample Size

The sample size was estimated based on knee VAS pain, using the formula n_1_ = n_2_ = 2 × [(Z_α_+Z_β_) × σ/δ],^2^ where an α level of .05 (two-sided; Z_α_ = 1.96) and a power of 80% (Z_β_ = 0.842) were applied. Assuming that the difference in VAS pain change between the 2 groups reaches the minimum clinically important difference (MCID) of 19.9 mm (δ) for individuals with knee OA,^32^ and considering an SD of 24.2 mm (σ) for the VAS pain from our previous trial,^33^ the calculated sample size (n_1_ = n_2_) is at least 24 for detecting a clinically significant reduction in knee VAS pain with glucocorticoid injection compared with saline injection. Accounting for a potential 20% loss to follow-up, a total sample size of 30 patients in each group was set as the recruitment target.

Since an MCID for effusion synovitis volume has not been defined, the detectable difference between the treatment and placebo groups was calculated based on a sample size of 30 per group and an SD (σ) of 7.69 mL, as observed in a previous trial.^34^ The calculated detectable difference is 6.22 mL.

### Statistical Analysis

The primary analyses involved intention-to-treat (ITT) analyses, encompassing all patients who provided informed consent and underwent randomization. The second analysis method was per-protocol (PP) analysis, which included only those who completed 4 visits without major protocol deviations.

A mixed-effects regression model for repeated measures was used to assess group differences in continuous outcomes across all available follow-up time points. The models included fixed effects for follow-up time, baseline covariates (age, sex, body mass index, baseline value of the corresponding outcome), treatment, and interactions between follow-up time and baseline covariates, as well as treatment. Adjustment for the corresponding baseline outcome was performed to control for baseline differences. Study site and individual participant identification were considered as the random intercepts to account for variability between participants and study site, while follow-up time served as the random slope in the models. The overall group differences were derived through the linear combination of the estimated coefficients. Because there were only 2 categories for pain medication use (increased and unchanged), a χ^2^ test was used to examine the difference in pain medication use between the treatment and placebo groups. The Fisher exact test was used to compare the incidence of adverse reactions between the 2 groups. Missing data were handled as described in the eMethods in Supplement 2. All analyses were performed using SAS version 9.4 (SAS Institute). A 2-sided *P* value of .05 was considered statistically significant.

## Results

### Participants

**Figure 1.**
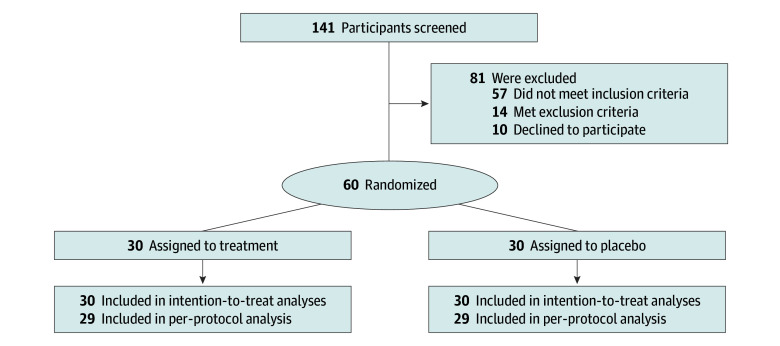
Flowchart of Participants in the GLITTERS Study GLITTERS indicates Glucocorticoid Injections Into the Infrapatellar Fat Pad in Patients With Knee Osteoarthritis.

Sixty patients were randomized and included in the ITT population (Figure 1), the mean (SD) age was 64.9 (11.1) years, and 38 participants (63.3%) were female. The baseline characteristics were well balanced between groups (Table 1). All participants completed the 4-, 8-, and 12-week follow-up. However, 1 to 2 participants had missing values in 1 to 3 items for the WOMAC score, 4-dimensional Assessment of Quality of Life (AQoL-4D), and PHQ-9, with all missing data following a nonmonotone pattern. A detailed summary of the sample sizes with complete data for each group and time point is provided in eTable 3 in Supplement 2. Given that 1 participant in each group exhibited significant deviations from the prescribed time windows, exceeding 4 weeks, there were 29 participants included in the PP analyses (Figure 1).

**Table 1.** Baseline Characteristics of Participants

Characteristic	Participants, mean (SD)		
	Total (N = 60)	Treatment (n = 30)	Placebo (n = 30)
Age, y	64.9 (11.1)	66.0 (10.8)	63.7 (11.4)
Sex, No. (%)			
Female	38 (63.3)	20 (66.7)	18 (60.0)
Male	22 (36.7)	10 (33.3)	12 (40.0)
Body mass index^a^	24.3 (2.6)	24.7 (2.6)	23.9 (2.5)
VAS, mm	65.0 (17.4)	66.0 (16.6)	64.0 (18.4)
Effusion synovitis volume, mL	18.8 (12.9)	20.0 (12.7)	17.6 (13.1)
WOMAC total score	942.0 (459.5)	911.5 (484.0)	972.5 (439.8)
AQoL-4D	82.9 (11.5)	81.7 (12.8)	84.0 (10.0)
Infrapatellar fat pad volume, ml	13.2 (3.6)	13.6 (3.9)	12.9 (3.2)
Hoffa synovitis			
1	0	0	0
2	20 (33.3)	11 (36.7)	9 (30.0)
3	40 (66.7)	19 (63.3)	21 (70.0)
WOMAC pain score	194.7 (106.2)	189.4 (104.4)	200.0 (109.6)
WOMAC stiffness score	62.5 (55.9)	53.7 (57.2)	71.3 (54.0)
WOMAC function score	684.8 (328.7)	668.4 (350.7)	701.2 (310.3)
PHQ-9	4.7 (4.5)	4.4 (4.4)	4.9 (4.7)
Bone marrow lesion, cm^2^	3.2 (3.9)	2.7 (3.6)	3.7 (4.2)
Cartilage defect	11.6 (2.5)	11.5 (2.7)	11.7 (2.4)
Effusion synovitis score			
1	11 (18.3)	7 (23.3)	4 (13.3)
2	38 (63.3)	15 (50.0)	23 (76.7)
3	11 (18.3)	8 (26.7)	3 (10.0)

Abbreviations: AQoL-4D, 4-dimensional Assessment of Quality of Life; PHQ-9, 9-item Patient Health Questionnaire; VAS, visual analog scale; WOMAC, Western Ontario and McMaster University Osteoarthritis Index.

^a^ Body mass index was calculated as weight in kilograms divided by height in meters squared.

### Primary Outcome Analyses

**Figure 2.**
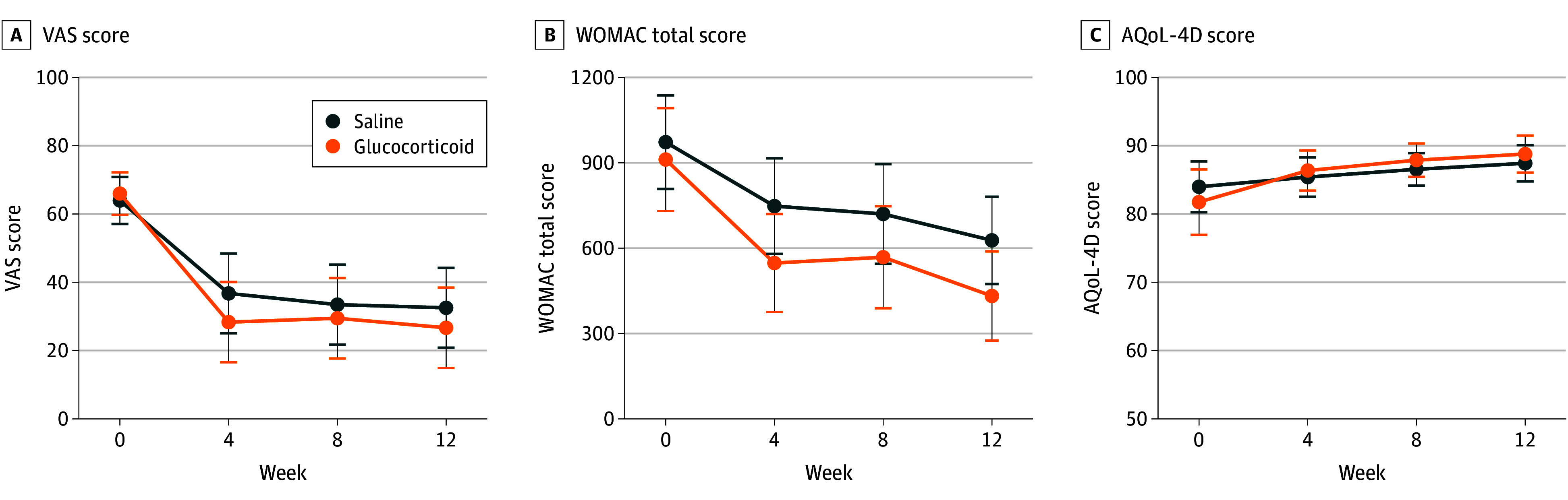
Intention-to-Treat Analysis of Changes in Outcomes With 4 Visits AQoL-4D indicates 4-dimensional Assessment of Quality of Life; VAS, visual analog scale; and WOMAC, Western Ontario and McMaster University Osteoarthritis Index.

In the ITT analysis, the treatment group exhibited an average reduction of 39.3 (95% CI, 27.5 to 51.1) points in VAS pain scores from baseline to 12 weeks, compared with a 31.4-point reduction (95% CI, 19.7 to 43.1 points) in the placebo group, resulting in a between-group difference of −7.9 points (95% CI, −19.7 to 4.0 points) (Table 2). Notably, the most significant decrease in VAS pain occurred within the first 4 weeks, with fewer changes in subsequent follow-ups (eTable 4 in Supplement 2; Figure 2). The within-participant and between-participant SDs for the VAS pain change were 12.4 and 20.5, respectively (eTable 5 in Supplement 2). The change in effusion synovitis volume from baseline to 12 weeks showed a reduction of 4.9 (95% CI, 2.0 to 7.8) mL in the treatment group vs a 5.4 (95% CI, 2.5 o 8.3) mL decrease in the placebo group, leading to a between-group difference of 0.5 mL (95% CI, −1.9 to 2.9 mL) (Table 2). PP analyses for the primary outcomes showed similar results (eTables 6 and 7 in Supplement 2).

**Table 2.** Intention-to-Treat Analysis of 12-Week Changes in All Outcomes Between the Treatment and Placebo Groups

Outcome	Least squares mean change (95% CI)		Between group difference in least squares mean change (95% CI)^a^	*P* value^b^
	Treatment (n = 30)	Placebo (n = 30)		
Primary outcomes				
VAS, mm	−39.3 (−51.1 to −27.5)	−31.4 (−43.1 to −19.7)	−7.9 (−19.7 to 4.0)	.19
Effusion synovitis volume, mL	−4.9 (−7.8 to −2.0)	−5.4 (−8.3 to −2.5)	0.5 (−1.9 to 2.9)	.67
Secondary outcomes				
WOMAC total	−479.6 (−636.3 to −322.8)	−345.2 (−498.4 to −192.0)	−134.4 (−353.0 to 84.2)	.22
AQoL-4D	7.0 (4.3 to 9.7)	3.4 (0.8 to 6.1)	3.6 (−0.2 to 7.4)	.06
Infrapatellar fat pad volume, mL	0.5 (0.2 to 0.8)	0.3 (−0.0 to 0.6)	0.2 (−0.2 to 0.6)	.37
Hoffa synovitis score	−0.2 (−0.5 to 0.1)	−0.1 (−0.4 to 0.2)	−0.1 (−0.3 to 0.2)	.46
Commenced or increased pain medication use, No. (%)^c^	3 (10.0)	9 (30.0)	NA	.05
Reporting ≥1 adverse reaction, No. (%)	1 (3.3)	1 (3.3)	NA	.99
Post hoc outcomes				
WOMAC pain score	−113.0 (−144.5 to −81.4)	−66.8 (−97.6 to −36.0)	−46.2 (−90.0 to −2.4)	.04
WOMAC stiffness score	−29.8 (−45.2 to −14.3)	−24.7 (−39.7 to −9.6)	−5.1 (−26.8 to 16.6)	.64
WOMAC function score	−343.9 (−461.0 to −226.9)	−248.1 (−362.6 to −133.6)	−95.8 (−259.0 to 67.4)	.24
PHQ-9 score	−2.6 (−4.5 to −0.7)	−1.8 (−3.7 to 0.1)	−0.8 (−2.5 to 0.9)	.35
Bone marrow lesion, cm^2^	−0.4 (−1.0 to 0.2)	−0.3 (−0.9 to 0.3)	−0.1 (−0.7 to 0.5)	.79
Cartilage defect	−0.1 (−1.1 to 0.8)	0.4 (−0.5 to 1.4)	−0.5 (−1.0 to −0.1)	.03
Effusion synovitis score	−0.2 (−0.4 to −0.0)	−0.2 (−0.4 to −0.0)	−0.0 (−0.3 to 0.2)	.81

Abbreviations: AQoL-4D, 4-dimensional Assessment of Quality of Life; PHQ-9, 9-item Patient Health Questionnaire; NA, not applicable; VAS, visual analog scale; WOMAC, Western Ontario and McMaster University Osteoarthritis Index.

^a^ Between-group differences in least squares mean change with 95% CI were derived from mixed-effects models, adjusted for follow-up time, baseline covariates (age, sex, body mass index, baseline value of the corresponding outcome), treatment, and interactions between follow-up time and baseline covariates as well as treatment.

^b^ The χ^2^ test or Fisher exact test was used.

^c^ Pain medication use was categorized as commenced or increased, unchanged, or discontinued or decreased. No participants in either group discontinued or decreased their medication.

### Secondary Outcome Analyses

No significant differences were observed between the treatment and placebo groups for the secondary outcomes. Regarding the WOMAC scores, the mean change from baseline at 12 weeks was −479.6 (95% CI, −636.3 to −322.8) points in the treatment group and −345.2 (95% CI, −498.4 to −192.0) points in the placebo group, resulting in a between-group difference of −134.4 (95% CI, −498.4 to 84.2) points (Table 2). For the AQoL-4D scores, the overall mean change from baseline in AQoL-4D scores was 7.0 (95% CI, 4.3 to 9.7) points in the treatment group compared with 3.4 (95% CI, 0.8 to 6.1) in the placebo group, with a between-group difference of 3.6 (95% CI, −0.2 to 7.4) (Table 2). Similar to the primary outcomes, the most notable increases in AQoL-4D and decreases in WOMAC scores occurred within the first 4 weeks (eTable 4 in Supplement 2; Figure 2). With regard to infrapatellar fat pad volume and Hoffa synovitis scores, no significant differences were observed between the groups. The between-group difference was 0.2 (95% CI, −0.2 to 0.6) mL for infrapatellar fat pad volume and −0.1 (95% CI, −0.3 to 0.2) for Hoffa synovitis scores. In terms of pain medication use, fewer participants in the treatment group (3 participants) required a dose increase compared with the placebo group (9 participants), while others maintained their doses (eTable 8 in Supplement 2). However, this difference did not reach statistical significance (*P* = .05) (Table 2). Both groups had 1 participant who experienced worse pain in the study knee, which was viewed as an adverse reaction. PP analyses for the secondary outcomes showed similar results (eTables 6 and 7 in Supplement 2).

### Post Hoc Analyses

In post hoc analyses, participants in the treatment group demonstrated greater improvements in symptoms (including WOMAC pain, stiffness, function, and PHQ-9 scores) compared with those in the placebo group, although most of these differences did not reach statistical significance (Table 2; eFigure in Supplement 2). Notably, the treatment group showed a statistically significant reduction in WOMAC pain scores, with a between-group difference of –46.2 (95% CI, –90.0 to –2.4). A significant improvement was also observed in cartilage defect scores, with a between-group difference of –0.5 (95% CI, –1.0 to –0.1) in favor of the treatment group. No significant between-group differences were found for bone marrow lesions or effusion synovitis scores. PP analyses yielded similar findings for the post hoc outcomes (eTables 6 and 7 in Supplement 2).

### Adverse Events

In the treatment group, 3 of 30 participants (10.0%) experienced at least 1 adverse event, compared with 7 of 30 (23.3%) in the placebo group. Detailed adverse events are shown in eTable 9 in Supplement 2.

## Discussion

GLITTERS, a 12-week multicenter, randomized, double-blind, placebo-controlled study, assessed the efficacy of glucocorticoid injection into the IPFP for alleviating knee pain and reducing effusion synovitis volume in individuals with inflammatory knee OA. The results indicated that the injection did not significantly reduce knee pain or effusion synovitis volume. Nevertheless, the treatment group showed improvement in other outcomes including symptoms, quality of life, and cartilage defects.

Intra-articular corticosteroid injections have been widely used for the management of local joint inflammatory symptoms.^18,19^ However, concerns about intra-articular corticosteroids have arisen due to findings from observational studies indicating adverse joint outcomes^35^ and a recent RCT highlighting cartilage loss.^23^ Given that the IPFP and synovium are key functional units in inflammation, administering a glucocorticoid injection directly into the IPFP may enhance efficacy and minimize side effects.

Regarding the primary outcomes of this trial, we observed a reduction of 7.9 points in VAS pain in the treatment group compared with the placebo group over 12 weeks, although this difference did not reach statistical significance. While intra-articular glucocorticoid injections are recommended in some guidelines, their effectiveness in pain relief remains controversial, with some studies indicating no greater efficacy than placebo.^1,23,36,37^ Moreover, a 24-month trial reported that knee VAS pain reduction was greater in the intra-articular placebo injection group than in the triamcinolone injection group at 3 months.^23^ In clinical practice, glucocorticoid injection into the IPFP may be more appropriate than intra-articular injection. Further clinical trials comparing the effects of intra-articular injections and IPFP injections of glucocorticoid on knee pain are needed. For the primary outcome of effusion synovitis volume, both groups showed comparable reductions, with no significant difference between the treatment and placebo groups.

For the secondary outcomes, the treatment group generally showed favorable results. Of note, 3 participants in the treatment group and 9 in the placebo group increased their pain medication dosage, although the between-group difference was not statistically significant. This in turn may also have compromised the comparative efficacy of IPFP glucocorticoid injections vs IPFP saline injections in knee pain to some extent. Regarding the potential adverse effects of administering glucocorticoids to the IPFP, both groups experienced 1 adverse reaction. Additionally, IPFP shrinkage remains a concern due to the potential for soft-tissue atrophy associated with local corticosteroid injections.^38,39^ However, our findings showed no evidence of IPFP shrinkage, and no significant difference in IPFP volume was observed between the groups. For the MRI assessments, we did not observe a statistically significant difference in Hoffa synovitis between the groups. Aside from the short follow-up period, another possible explanation is that we used a noncontrast MRI. While noncontrast MRI is commonly used in clinical practice, the use of contrast-enhanced MRI could have provided clearer visualization of synovitis. This may have improved the sensitivity in detecting inflammation over time, particularly in a short follow-up period. Moreover, synovitis from contrast-enhanced MRI could facilitate more precise participant selection, identifying those more likely to respond to the intervention and thereby enhancing the accuracy of participant inclusion.

Regarding the post hoc analysis outcomes, we observed a statistically significant between-group difference in WOMAC pain scores, with a standardized mean difference (SMD) of −0.54. Although this indicates a potential moderate treatment effect according to Cohen *d*, it remains lower than the SMD equivalent of the MCID, which is approximately 0.92 for WOMAC pain.^40^ However, the observed effect size exceeded that of intra-articular glucocorticoid injection at 13 weeks (SMD, −0.22),^22^ implying a potentially superior efficacy of the intra-IPFP route. This potential advantage warrants confirmation in future head-to-head clinical trials directly comparing intra-articular and intra-IPFP glucocorticoid injections. Notably, a significant reduction in cartilage defects was also observed in the treatment group compared with the placebo group (between group difference, 0.5). In our previous study, participants with higher total cartilage defect scores were 6 times more likely to undergo total knee replacement than those with lower scores over the subsequent 4 years.^41^ Assuming a log-linear relationship between cartilage defect and knee replacement risk, and considering that the IQR at baseline in this trial was 5 (approximating the high vs low group difference), a 0.5-unit difference in cartilage defect corresponds to an estimated odds ratio of approximately 1.2 for knee replacement. However, given the limited sample size, the possibility that this finding arose by chance cannot be excluded. Further large-scale and long-term studies are needed to confirm whether corticosteroid injection into the IPFP confers structural benefits to cartilage.

### Strengths and Limitations

An important strength of our trial is that we specifically targeted participants with inflammatory OA characterized by effusion synovitis and Hoffa synovitis, as assessed by MRI. Another strength is that we had high adherence and no loss to follow-up.

However, this study also has limitations. First, while the 12-week duration was adequate for evaluating inflammatory and symptomatic outcomes, it was insufficient to assess structural changes, such as IPFP atrophy and cartilage loss, which would require a longer follow-up period. Second, it remains unclear whether glucocorticoid injection into the IPFP can diffuse into the joint cavity, potentially leading to direct contact with the cartilage and causing adverse effects similar to those caused by intra-articular glucocorticoid injections. Third, the sample size was determined based on the MCID, which is often an optimistic estimate of treatment effect in knee OA trials. As a result, the study may have been underpowered to detect more modest but clinically relevant differences between groups. Future studies with larger sample sizes may provide more robust evidence. Fourth, although intra-articular hyaluronic acid injections were administered equally in both groups, patients’ awareness of receiving a potentially active therapy may have enhanced placebo effects, potentially influencing the perceived benefits of the treatment under investigation. Fifth, this study included only participants with inflammatory features of knee OA; therefore, the observed effects of intra-articular glucocorticoid injections may not be generalizable to patients with fewer inflammatory features or noninflammatory OA phenotypes. Furthermore, the study design did not permit a direct comparison between IPFP and intra-articular glucocorticoid injections, thereby limiting our ability to assess the relative benefits and risks of these 2 approaches. Future clinical trials directly comparing intra-articular and IPFP glucocorticoid injections are warranted to clarify their respective efficacy and safety profiles.^42^

## Conclusions

In conclusion, among patients with knee OA and effusion synovitis and Hoffa synovitis, glucocorticoid injections into the IPFP did not significantly alleviate pain or reduce effusion synovitis volume over 12 weeks. However, as certain post hoc outcomes favored glucocorticoid injections, further investigation may be helpful.

## Notes

Supplement 1.
Trial Protocol and Statistical Analysis Plan

Supplement 2.
eMethods.
**eTable 1.** Magnetic Resonance Imaging Sequences and Parameters Across the 4 Study Sites **eTable 2.** Intrareader and Interreader Reliability and Smallest Detectable Change for MRI Outcomes **eTable 3.** Sample Sizes of Participants With Complete Data for Each Group at Each Follow-Up Time Point **eTable 4.** Detailed Results for Intention-to-Treat Analyses Using Mixed-Effect Model **eTable 5.** Within- and Between-Participant Standard Deviations for Outcomes Using Mixed Model for Repeated Measures **eTable 6.** Per-Protocol Analysis of 12-Week Changes in All Outcomes Using Mixed-Effects Regression **eTable 7.** Detailed Results for Per-Protocol Analyses Using Mixed-Effect Model **eTable 8.** Pain Medication Use Details in Participants Reporting Increased Use **eTable 9.** Adverse Events **eFigure.** Intention-to-Treat Analysis of Changes in Post Hoc Outcomes With 4 Visits: WOMAC Pain, WOMAC Stiffness, WOMAC Function, and PHQ-9

Supplement 3.
Data Sharing Statement
