# Supplementary material for: Infrapatellar Fat Pad Glucocorticoid Injection in Knee Osteoarthritis: A Randomized Clinical Trial
Source: JAMA Netw Open. 2026 Jan 2;9(1):e2549938. doi: 10.1001/jamanetworkopen.2025.49938 (PMC12761338; doi:10.1001/jamanetworkopen.2025.49938)
Supplement: Supplement 1. — Trial Protocol and Statistical Analysis Plan [file jamanetwopen-e2549938-s001.pdf]

Efficacy and safety of GLucocorticoid injections into InfrapaTellar faT pad in patients with knee ostEoarthRitiS: protocol for the GLITTERS randomized controlled trial

The principal study center: Zhujiang Hospital of Southern Medical University

Principal Investigator: Changhai Ding

Version: Version 2

Date: 2022/2/11

Confidentiality Statement

All information contained in this research protocol is the property of the researchers of this project and is provided solely for review by the Ethics Committee and relevant institutions. Without the written consent of the Principal Investigator (PI), it is strictly prohibited to disclose any information to third parties not involved in this research.

Protocol Confirmation Signature Page

Principal Investigator's Consent to the Protocol:

I have carefully read this protocol, agree with all its contents, and consent to execute it as described in the protocol.

I understand that the study cannot commence without the approval of the Ethics Committee; I understand that informed consent and corresponding documentation must be obtained from all participants involved in the study; I understand that the study must be conducted in accordance with the ethical principles outlined in the Helsinki Declaration and relevant clinical research laws and regulations; I understand that all relevant regulations of our institution must be fully complied with.

Name: Changhai Ding

Signature: 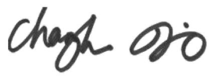

Date: 2022/2/11

Abstract

Background

Knee osteoarthritis (OA) is a prevalent disabling disorder that involves changes in articular cartilage damage, subchondral bone remodeling, synovitis, and abnormal infrapatellar fat pad (IPFP). Due to the complicated etiology and numerous phenotypes of knee OA, limited improvement is achieved for treatments among knee OA patients with different phenotypes. Inflammatory OA phenotype is a typical knee OA phenotype, and individualized treatment targeting on inflammation is a promising way to obtain an optimal therapeutic effect for people with inflammatory knee OA phenotype. Glucocorticoid is a traditional anti-inflammatory drug for knee OA and intra-articular glucocorticoid injections are recommended clinically. However, emerging evidence has shown the repeated intra-articular glucocorticoid injections for a long term would induce cartilage loss. IPFP and its adjacent synovium are considered as the main source of inflammation in knee OA. This GLITTERS trial aims to investigate if a glucocorticoid injection into the IPFP is effective and safe over 12 weeks among knee OA patients with an inflammatory phenotype.

Methods

GLITTERS is a multicenter, double-blinded, randomized, and placebo-controlled clinical trial among knee OA patients with both Hoffa-synovitis and effusion-synovitis. Sixty participants will be allocated randomly and equally to either the glucocorticoid group or the control group. Each group will receive an injection of glucocorticoid or saline into the IPFP with an intra-articular hyaluronic acid injection as a background treatment at baseline, and be followed 4, 8, and 12 weeks. Primary outcomes will be changes in knee pain on a visual analogue scale and effusion-synovitis volume measured on magnetic resonance imaging (MRI). Secondary outcomes will be changes in the total score of Western Ontario and McMaster Universities Osteoarthritis Index score, MRI-detected Hoffa-synovitis score, quality of life, pain medication use, IPFP

volume, and the incidence of adverse reactions. Data analyses based on the intention-to-treat principle will include mixed-effects regressions, Wilcoxon rank-sum tests, and chi-square tests (or Fisher's exact test).

Introduction

Osteoarthritis (OA) is a highly prevalent joint disease characterized by joint pain and structural changes which ultimately lead to loss of joint function[1]. According to the estimation of the World Health Organization, there are about 300 million OA patients worldwide, and the prevalence of OA among people over 50 years old can reach up to 10-20%[2, 3]. Knee OA is the predominant type of OA whose pathological changes include articular cartilage damage, subchondral bone remodeling, synovitis, and abnormal infrapatellar fat pad (IPFP, or Hoffa's fat pad)[4]. Knee OA seriously decreases the quality of life of the patients and leads to a heavy economic burden to patients' families as well as society[1, 4].

However, there is no curative drug for knee OA. Apart from some patients with end-stage knee OA undergoing total knee arthroplasty, most patients choose conservative treatments to alleviate their symptoms[4]. Due to the complicated etiology and numerous subtypes of knee OA, limited improvement is achieved for similar treatments among patients with different knee OA phenotypes[4]. Therefore, an individualized treatment focusing on a specific phenotype of knee OA is a promising way to obtain an optimal therapeutic effect.

Inflammatory phenotype is one of the most typical phenotypes of knee OA[5-7]. IPFP and synovium as a structural complex are considered as the main source of inflammation in knee OA[8]. IPFP, an adipose tissue, is below the patella and located closely to the synovial layers[9]. Adipocytes and immune cells are abundant in IPFP[9]. Abnormal IPFP can release a variety of inflammatory products which result in changes of cartilage, synovium, and subchondral bone, and eventually accelerate OA progression[10]. Signal changes in IPFP detected by magnetic resonance imaging (MRI)

are referred to as Hoffa-synovitis[11]. Effusion-synovitis, a composite of joint fluid and synovial thickening, refers to the MRI-detected signal changes in the synovial cavity. Abundant clinical evidence showed that Hoffa-synovitis and effusion-synovitis were associated with the incidence and progression of knee OA[11-18]. Therefore, anti-inflammatory therapies for those knee OA patients with Hoffa- and effusion-synovitis may have a better clinical effect.

Glucocorticoid is a traditional anti-inflammatory drug for knee OA. It suppresses inflammation via the apoptosis induction of immune cells and the suppression of the proinflammatory mediators expression[19]. Clinically, intra-articular glucocorticoid injections are recommended for people with knee OA to reduce their pain[20, 21]. However, emerging evidence has shown that repeated intra-articular injections over two years would induce more cartilage loss [22]. Thus, the injection of glucocorticoid into IPFP may not only play a better role in anti-inflammatory but minimize the cartilage deterioration among inflammatory knee OA patients. To date, there is no study about the injection of drugs into IPFP.

The characteristics of real-time imaging and tracking in ultrasonography make the injection into inflammatory sites of IPFP possible[23, 24]. Therefore, we hypothesized that the IPFP injection of glucocorticoid can effectively reduce the knee pain and effusion--synovitis volume, compared to the placebo injection among people with symptomatic knee OA who have effusion- and Hoffa-synovitis.

Objectives

The primary aim is to assess whether the injection of glucocorticoid into the IPFP can effectively reduce the knee pain measured by visual analogue scale (VAS) and MRI-measured effusion-synovitis volume, compared to the placebo injection among patients with symptomatic knee OA who have both effusion- and Hoffa-synovitis.

Secondary aims are to evaluate whether the injection of glucocorticoid into the IPFP effectively reduces the total Western Ontario and McMaster Universities Osteoarthritis Index (WOMAC) score, MRI-assessed Hoffa-synovitis score and pain

medication use, improves the quality of life, and has difference in IPFP volume and adverse reaction, compared to the placebo injection among symptomatic knee OA patients with both effusion- and Hoffa-synovitis.

Methods and analysis

Study design

GLITTERS is a multicenter, randomized, double-blind, placebo-controlled trial over 12 weeks (figure 1). The ethics approval in all four centers has been received from the Medical Ethics Committee before the recruitment. The principal study center is Zhujiang Hospital of Southern Medical University (2021-KY-183-02), and the other study centers are the Sixth Affiliated Hospital of Sun Yat-sen University (2022ZSLYEC-179), the Third Affiliated Hospital of Sun Yat-sen University ([2022]02-138-01), and Beihai People's Hospital (2022-014). This study will use competitive enrollment, and all participants will provide informed written consent prior to data collection. The trial will be reported in accordance with the Consolidated Standards of Reporting Trials guidelines.

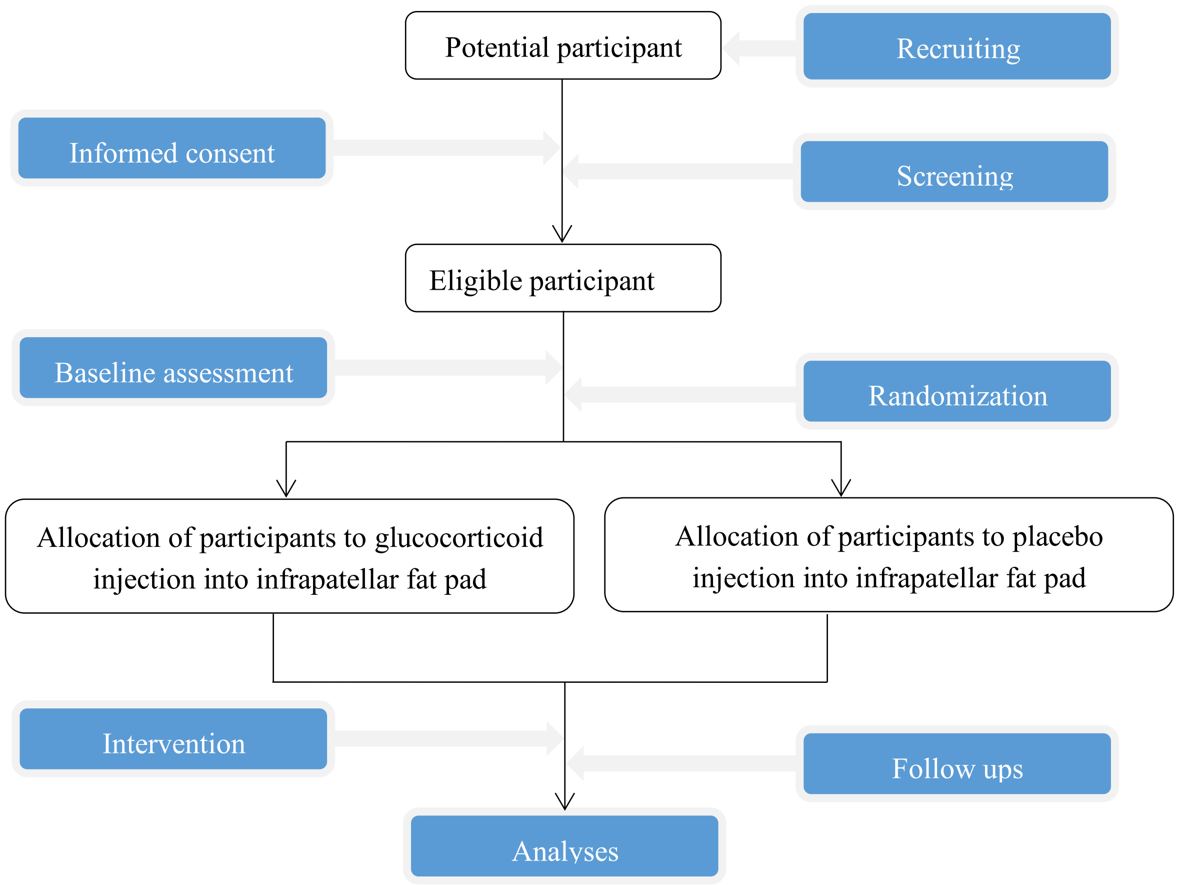

Figure. Flowchart of the trial.

Recruitment and informed consent

The potential participants will be recruited in the following ways: 1) referrals by the participating doctors from outpatient clinics; 2) public education from the surrounding community hospitals; 3) posters/flyers placed on hospitals and surrounding communities; and 4) electronic advertisements on social media platforms. Each participant will be asked to sign two copies of the informed consent to ensure that their participation is voluntary. One copy is preserved by the principal researcher, and the other by the participant.

Eligibility criteria

Inclusion criteria:

1) Diagnosed with symptomatic knee OA according to American College of Rheumatology criteria[25];
2) Age > 45 years;
3) Have knee pain for more than six months and the pain assessed by VAS (100 mm) ≥ 40 mm in last week;
4) Ultrasonography showed obvious synovitis (over about 10 ml) with effusion in the knee joint;
5) Both MRI-assessed Hoffa-synovitis score (MRI Osteoarthritis Knee Score, MOAKS method)[26, 27] and effusion-synovitis score (MOAKS method)[26] ≥ 1, and their total score ≥ 3;
6) Being able to listen, speak, read and understand Chinese, capable of understanding the study requirements and cooperating with the researchers during the study, and providing written informed consent.

Exclusion criteria

1) Allergy to glucocorticoids;
2) Knee joint injection of glucocorticoid or hyaluronic acid within the past six months;
3) Severe trauma or arthroscopy in the knee within the past six months;
4) Planned hip or knee surgery (including arthroscopy, arthroplasty, and other open joint surgeries) in the next six months;
5) Contraindication to having MRI (e.g., implanted pacemaker, artificial metal valve or cornea, aneurysm clipping surgery, arterial dissection, metal foreign bodies in the eyeball, claustrophobia);
6) Presence of other arthritis, such as rheumatoid arthritis and psoriatic arthritis;
7) Other condition that is more painful than their knee OA;
8) Malignant tumors or other life-threatening diseases;
9) Infection, diabetes, coagulopathy, osteonecrosis, or gastric/duodenal ulcer within the past 12 months;
10) Current use of oral corticosteroids, nonsteroidal anti-inflammatory drugs, or immunosuppressive medication;
11) Pregnancy or lactating female;
12) Use any investigational drugs or devices in the recent 30 days.

Note: When both knees of the participants meet the eligibility criteria, the knee with more severe VAS pain will be selected as the study knee.

Randomization and blinding

Using block randomization in block size of four, eligible participants in each center will be assigned to the treatment or placebo group with a 1:1 allocation rate based on computer-generated random numbers. A staff member who is not involved in this trial will pack the drugs with a random number indicated on each of the packages. Allocation concealment will be ensured and Allocation results will be not revealed until the completion of final data analyses. Double-blind (participants & researchers

including outcome assessors and statisticians) design will be applied in this trial. Because of the different appearance of the injection drugs, the physical therapists for the injections are not blinded, and they will not be involved in other processes of this trial. During the period of preparing and injecting the drug, participants will be out of sight of the injection drugs. Unblinding will be conducted after all the data analyses are obtained. Emergency unblinding will be permissible when a serious adverse event happens, and patients who are unblinded will be withdrawn from the trial.

Intervention

Participants are randomly allocated to either treatment (glucocorticoid) group or placebo (saline) group. Each group will receive two injections at baseline: one is the glucocorticoid or saline into IPFP, and the other is intra-articular hyaluronic acid as a background treatment.

The product of the glucocorticoid is betamethasone injectable suspension (Diprosone) and the dosage is 1 ml. To alleviate the discomfort on local tissues, the suspension injected into the IPFP will be pre-mixed with 0.5 ml saline and 0.5 ml lidocaine (concentration: 2%). That is, the total amount of the drug injected into the IPFP for the glucocorticoid group will be 2.0 ml. Accordingly, a total of 2.0 ml drug including 1.5 ml saline and 0.5 ml lidocaine will be injected into the IPFP in the placebo group. The injection of glucocorticoid or placebo into the IPFP will be performed under the guidance of ultrasonography. The procedures are as follows: 1) the participant lies on the bed with a small pillow behind his/her knee to bend the knee joint by 20-30°; 2) the ultrasonic probe is placed under the patella to show the IPFP and its inflammatory sites (figure 1); 3) insert the needle through the inferior lateral patella; 4) the drug is injected on two sites at the bottom of IPFP near the synovium where synovial hyperplasia is obvious, with a dosage of 1 ml at each site. All physical therapists will be trained for this injection under the supervision of an experienced therapist.

The intra-articular hyaluronic acid (ARTZ, Seikagaku Corporation, Japan) injection will be added as a background treatment. That is, after completing the process

of IPFP injection, the participants in both groups will receive 2.5 ml hyaluronic acid suspension injection through the suprapatellar bursa into the intra-articular space under the guidance of ultrasonography.

Outcome measures

An overview of the data collection is listed in Table 1. All questionnaires (VAS, WOMAC, four-dimensional Assessment of Quality of Life: AQoL-4D, and nine-items Patient Health Questionnaire: PHQ-9) and queries about pain medication use and the use of medication & supplements will be applied at baseline, 4, 8, and 12 weeks follow-ups. MRI assessments including effusion-synovitis volume, Hoffa-synovitis score, tibiofemoral bone marrow lesion maximum size, tibiofemoral cartilage defects, and IPFP volume will be examined at baseline and 12 weeks follow-up. Other measures including demographic data, clinical evaluation and history collection, ultrasonographic examination in the study knee, and effusion-synovitis score assessed by MRI will be recorded at baseline/screening only.

Table 1. Schedule of data collection

	**Screening/ Baseline**	**4 weeks***	**8 weeks***	**12 weeks**
**Primary outcomes**				
Knee pain (VAS)	✓	✓	✓	✓
Effusion-synovitis volume#	✓			✓
**Secondary outcomes**				
WOMAC	✓	✓	✓	✓
Hoffa-synovitis score# (MOAKS)	✓			✓
Quality of life (AQoL-4D)	✓	✓	✓	✓
Pain medication use	✓	✓	✓	✓
infrapatellar fat pad volume#	✓			✓

Adverse reaction		✓	✓	✓
**Other measures**				
Demographic data†	✓			
Clinical evaluation§ and history collection	✓			
Ultrasonographic examination in the study knee	✓			
Effusion-synovitis score# (MOAKS)	✓			✓
Tibiofemoral bone marrow lesion maximum size#	✓			✓
Tibiofemoral cartilage defect#	✓			✓
Use of medication & supplements	✓	✓	✓	✓
Adverse events		✓	✓	✓
Depression (PHQ-9)	✓	✓	✓	✓

VAS: visual analogue scale; WOMAC: Western Ontario and McMaster Universities Osteoarthritis Index; MOAKS: MRI Osteoarthritis Knee Score; AQoL-4D: four-dimensional Assessment of Quality of Life; PHQ-9: nine-items Patient Health Questionnaire.

* The participants will complete the questionnaires (VAS, WOMAC, AQoL-4D, an PHQ-9) of the follow-ups at home and send back to researchers. As for pain medication use, use of medication & supplements, and adverse events, the researchers will ask the participants via remote interview.

# In MRI.

† Including names, gender, birth date, height, weight and contact details.

§ Diagnosis of symptomatic knee osteoarthritis according to American College of Rheumatology criteria.

Questionnaires and queries

The VAS (100 mm visual analog scale)[28] will be used to assess knee pain using the standard question: "On this line, how would you rate your knee pain in the last week?". A higher score indicates a higher level of pain severity.

The WOMAC[29] requires patients to rate their pain (five items), stiffness (two items), and functional dysfunction (17 items) in the last week. Each item is a 100-mm visual analog scale. The WOMAC score is calculated by summing the score of each

item with every 1 mm representing one point. A higher WOMAC score indicates a more severe OA symptom. When more than five items are not completed, the WOMAC score would be regarded as missing data. Otherwise, the WOMAC score would be calculated by averaging the remaining score and then multiplying by 24.

The AQoL-4D[30] will be used to assess the quality of life in the last week. It comprises four dimensions of independent living, social relationships, psychological well-being, and physical senses. Each dimension has three items with four response categories ranging from 0-3. The total AQoL-4D score ranges from 0-36, and a higher score indicates a lower quality of life.

The PHQ-9[31] will be used to assess the depression in the last two weeks. It comprises nine items with four response categories scored from 0-3. The total PHQ-9 score ranges from 0-27, and a higher score indicates a more severe depression level.

Queries about medications and supplement use will also be recorded at baseline and each follow-up. Adverse events and reactions will be recorded at each follow-up. Pain medication use would be determined by the researchers from the records of medication use. Adverse events are defined as any untoward event occurring during the trial, regardless of its relation to treatment, and they need to be reported spontaneously when they occur. Details of the adverse event will be recorded and whether it is an adverse reaction will be determined.

MRI assessments

Sagittal images on intermediate-weighted/proton density-weighted fat suppression sequences will be used in assessing effusion-synovitis volume/score, Hoffa-synovitis score, tibiofemoral bone marrow lesion maximum size, tibiofemoral cartilage defects, and IPFP volume.

Effusion-synovitis volume will be calculated by summing the volume of effusion synovitis in suprapatellar pouch, central portion, posterior femoral recess, and submuscular recess using OsiriX software. Effusion-synovitis score was scored using MOAKS which can be divided into 0-3 grades[26].

Hoffa-synovitis score (MOAKS method) will be assessed according to the discrete area with increased signal intensity in the IPFP on MRI images[26, 27]. It can be divided into 0-3 grades: grade 0=normal, grade 1=IPFP with an increase of signal intensity of <10%, grade 2=IPFP with an increased signal intensity between 10% and 20%, grade 3=IPFP with an increase in signal intensity of >20%.

Tibiofemoral bone marrow lesions are defined as discrete areas of increased signal in the subchondral bone. Bone marrow lesions maximum size will be assessed at the medial tibial, medial femoral, lateral tibial, and lateral femoral compartments. Slice with the greatest area of bone marrow lesions in a specific compartment will be chosen to assess bone marrow lesions' maximum size of the corresponding compartment. Bone marrow lesions on adjacent slices will be measured and compared to locate the slice with the maximum lesion size[32]. Tibiofemoral bone marrow lesions' maximum size will be calculated by summing the maximum lesions' size of the four compartments.

Cartilage defects will be graded using a modified Outerbridge classification as follows: grade 0=normal, grade 1=focal blistering and intra-cartilaginous hyperintensity with a normal contour, grade 2=irregularities on the surface and loss of thickness of less than 50%, grade 3=deep ulceration with loss of thickness of more than 50% without exposure of subchondral bone, grade 4=full-thickness chondral wear with exposure of subchondral bone[33]. Cartilage defects will be assessed at the medial tibial, medial femoral, lateral tibial, and lateral femoral compartments, and tibiofemoral cartilage defects will be obtained by summing the scores of the four compartments.

IPFP volume will be measured by manually drawing disarticulation contours around the IPFP boundaries using OsiriX software as reported previously[34].

Data management

Research Electronic Data Capture (REDCap), a secure web-based application, will be applied to facilitate the data collection throughout the study. It has a function of self-monitoring in data entry which maximizes data quality. After each follow-up, the researcher will timely input the obtained data into REDCap, and a backup REDCap

data will be regularly stored at the principal study center. At the completion of the follow-up, the MR images will be assessed. After all the data are obtained, unblinding will be undertaken. In this trial, the two-step unblinding method will be applied. In the first step of unblinding, the participants will be classified into group A and group B for data analyses. After that, the second step of unblinding will assign the two groups to the treatment group or placebo group.

The participants are allowed to withdraw at any time throughout the study. If the participants withdraw from the study before the end of the trial, they are asked to have data collected including MRI assessment. The reason and date of the withdrawal will be recorded and the data before the withdrawal will be asked to retain.

Sample size

We estimated the sample size based on the primary outcomes. The formula $n_1=n_2=2\times[(Z_\alpha+Z_\beta)\times\sigma/\delta]^2$ was used to calculate the sample size with an α level of 0.05 (two-sided; $Z_\alpha=1.96$) and a power of 80% ($Z_\beta=0.842$). It is reported that the minimum clinically important difference (MCID) of knee VAS pain in people with knee OA is 19.9 mm. Assuming that the knee VAS pain reduction in the glucocorticoid injection group compared with the saline injection group is clinically significant, the difference between knee VAS pain changes of the two groups should be at least 19.9 mm (δ). As the standard deviation of the change in knee VAS pain from baseline to three months follow-up is 24.2 mm (σ) in our previous trial[35], the $n_1=n_2$ calculated is 24. Considering a 20% loss to follow-up, a sample size of 30 patients in each group is needed.

As an MCID for effusion-synovitis volume has not yet been defined, the detectable difference of effusion–synovitis volume between the treatment and placebo groups were calculated based on the given sample size ($n_1=n_2=30$) and σ. According to the standard deviation of the change in effusion-synovitis volume from baseline to 12 weeks follow-up being 7.69 ml (σ) in the same previous trial[36], the detectable difference of effusion–synovitis volume calculated is 6.22 ml.

Statistical plan

Baseline characteristics will be displayed in descriptive statistics according to the data type. Continuous variables with normal distribution will be displayed by means and standards deviation. Continuous variables with non-normal distribution will be displayed by median and interquartile range. Category variables will be displayed by proportion.

The intention-to-treat analysis will be the primary analyses method. The per-protocol analysis will be the second analyses method where the per-protocol population is defined as participants completed four follow-ups without major protocol deviations. There is no interim analysis in this research. Missing data due to dropout and nonresponses will be addressed using multiple imputations with chain equations. For each treatment group, imputations will be performed separately based on the baseline characteristics and the non-missing values at other time points of the missing variables with the assumption of data missing at random. Imputations will be performed separately for each treatment group and each outcome using baseline variables of age, sex, BMI, and study site and non-missing values of the outcomes at baseline and each follow-up with the assumption of data missing at random.

Mixed-effects regression models will be used to calculate treatment group differences with continuous measures. In the models, fixed effects will be follow-up time, baseline covariates (including age, sex, BMI, baseline value of the corresponding outcome), treatment, and interactions between follow-up time and baseline covariates and treatment. Study site and individual participant identification will be treated as the random intercepts and follow-up time as the random slop in the models. The overall treatment group differences will be calculated by the linear combination of the estimated coefficients. Wilcoxon rank-sum test will be used to analyze the difference in pain medication use between the treatment and placebo groups. Pain medication use will be classified as commenced/increased, unchanged, or discontinued/decreased. The chi-square test or Fisher's exact test will be used to compare the incidence of adverse reactions between the two treatment groups, and the number of adverse reactions will

be reported as the number of participants reporting at least one adverse reaction. All analyses will be performed using Stata, version 15 (StataCorp). A 2-sided *P* value of 0.050 will be treated as statistically significant.

Abbreviations

OA: osteoarthritis; IPFP: infrapatellar fat pad; MRI: magnetic resonance imaging; VAS: visual analogue scale; WOMAC: Western Ontario and McMaster Universities Osteoarthritis Index; MOAKS: MRI Osteoarthritis Knee Score; AQoL-4D: four-dimensional Assessment of Quality of Life; PHQ-9: nine-items Patient Health Questionnaire; REDCap: Research Electronic Data Capture; MCID: minimum clinically important difference.

Audits and steering

The Trial Steering Committee will meet PIs to review the trial conduct every 3 months. Each electronic case report form will be checked by a research coordinator for completion. This committee's task is to ensure the trial follows the study plan. As it is a low-risk intervention trial, a Data Monitoring Committee is not considered.

Protocol amendments

In case of any protocol amendments, the amended protocol will be submitted to Clinical Trial Governance Committee and Ethical Committee for approval. Each PI of the study center will then be notified for the amendments. A copy of the revised protocol will be added to the Investigator Site File. The amendments will be recorded in CinicalTrials.gov. Any deviations from the protocol will be fully documented using a breach report form.

References

1. Safiri S, Kolahi AA, Smith E, et al. Global, regional and national burden of osteoarthritis 1990-2017: a systematic analysis of the Global Burden of Disease Study 2017. *Annals of the rheumatic diseases* 2020; 79: 819-828. 2020/05/14. DOI: 10.1136/annrheumdis-2019-216515.
2. Blanco FJ, Valdes AM and Rego-Pérez I. Mitochondrial DNA variation and the pathogenesis

of osteoarthritis phenotypes. *Nature reviews Rheumatology* 2018; 14: 327-340. 2018/04/20. DOI: 10.1038/s41584-018-0001-0.
3. Global, regional, and national incidence, prevalence, and years lived with disability for 354 diseases and injuries for 195 countries and territories, 1990-2017: a systematic analysis for the Global Burden of Disease Study 2017. *Lancet (London, England)* 2018; 392: 1789-1858. 2018/11/30. DOI: 10.1016/s0140-6736(18)32279-7.
4. Hunter DJ and Bierma-Zeinstra S. Osteoarthritis. *Lancet (London, England)* 2019; 393: 1745-1759. 2019/04/30. DOI: 10.1016/s0140-6736(19)30417-9.
5. Deveza LA, Nelson AE and Loeser RF. Phenotypes of osteoarthritis: current state and future implications. *Clinical and experimental rheumatology* 2019; 37 Suppl 120: 64-72. 2019/10/18.
6. Dell'Isola A, Allan R, Smith SL, et al. Identification of clinical phenotypes in knee osteoarthritis: a systematic review of the literature. *BMC musculoskeletal disorders* 2016; 17: 425. 2016/10/14. DOI: 10.1186/s12891-016-1286-2.
7. Mobasheri A, Saarakkala S, Finnilä M, et al. Recent advances in understanding the phenotypes of osteoarthritis. *F1000Research* 2019; 8 2019/12/31. DOI: 10.12688/f1000research.20575.1.
8. Greif DN, Kouroupis D, Murdock CJ, et al. Infrapatellar Fat Pad/Synovium Complex in Early-Stage Knee Osteoarthritis: Potential New Target and Source of Therapeutic Mesenchymal Stem/Stromal Cells. *Frontiers in bioengineering and biotechnology* 2020; 8: 860. 2020/08/28. DOI: 10.3389/fbioe.2020.00860.
9. Favero M, El-Hadi H, Belluzzi E, et al. Infrapatellar fat pad features in osteoarthritis: a histopathological and molecular study. *Rheumatology (Oxford, England)* 2017; 56: 1784-1793. 2017/09/29. DOI: 10.1093/rheumatology/kex287.
10. Zeng N, Yan ZP, Chen XY, et al. Infrapatellar Fat Pad and Knee Osteoarthritis. *Aging and disease* 2020; 11: 1317-1328. 2020/10/06. DOI: 10.14336/ad.2019.1116.
11. Roemer FW, Guermazi A, Felson DT, et al. Presence of MRI-detected joint effusion and synovitis increases the risk of cartilage loss in knees without osteoarthritis at 30-month follow-up: the MOST study. *Annals of the rheumatic diseases* 2011; 70: 1804-1809. 2011/07/28. DOI: 10.1136/ard.2011.150243.
12. Atukorala I, Kwoh CK, Guermazi A, et al. Synovitis in knee osteoarthritis: a precursor of disease? *Annals of the rheumatic diseases* 2016; 75: 390-395. 2014/12/10. DOI: 10.1136/annrheumdis-2014-205894.
13. Driban JB, Harkey MS, Barbe MF, et al. Risk factors and the natural history of accelerated knee osteoarthritis: a narrative review. *BMC musculoskeletal disorders* 2020; 21: 332. 2020/05/31. DOI: 10.1186/s12891-020-03367-2.
14. Wang X, Blizzard L, Jin X, et al. Quantitative Assessment of Knee Effusion-Synovitis in Older Adults: Association With Knee Structural Abnormalities. *Arthritis & rheumatology (Hoboken, NJ)* 2016; 68: 837-844. 2015/12/05. DOI: 10.1002/art.39526.
15. Wang X, Blizzard L, Halliday A, et al. Association between MRI-detected knee joint regional effusion-synovitis and structural changes in older adults: a cohort study. *Annals of the rheumatic diseases* 2016; 75: 519-525. 2015/01/01. DOI: 10.1136/annrheumdis-2014-206676.
16. Wang X, Jin X, Han W, et al. Cross-sectional and Longitudinal Associations between Knee Joint Effusion Synovitis and Knee Pain in Older Adults. *The Journal of rheumatology* 2016; 43: 121-130. 2015/11/17. DOI: 10.3899/jrheum.150355.

17. MacFarlane LA, Yang H, Collins JE, et al. Association of Changes in Effusion-Synovitis With Progression of Cartilage Damage Over Eighteen Months in Patients With Osteoarthritis and Meniscal Tear. *Arthritis & rheumatology (Hoboken, NJ)* 2019; 71: 73-81. 2018/08/23. DOI: 10.1002/art.40660.
18. Abbasi B, Pezeshki-Rad M, Akhavan R, et al. Association between clinical and sonographic synovitis in patients with painful knee osteoarthritis. *International journal of rheumatic diseases* 2017; 20: 561-566. 2016/02/26. DOI: 10.1111/1756-185x.12834.
19. Savvidou O, Milonaki M, Goumenos S, et al. Glucocorticoid signaling and osteoarthritis. *Molecular and cellular endocrinology* 2019; 480: 153-166. 2018/11/18. DOI: 10.1016/j.mce.2018.11.001.
20. Kolasinski SL, Neogi T, Hochberg MC, et al. 2019 American College of Rheumatology/Arthritis Foundation Guideline for the Management of Osteoarthritis of the Hand, Hip, and Knee. *Arthritis & rheumatology (Hoboken, NJ)* 2020; 72: 220-233. 2020/01/08. DOI: 10.1002/art.41142.
21. Practitioners RACoG. Guideline for the management of knee and hip osteoarthritis, https://www.racgp.org.au/download/Documents/Guidelines/Musculoskeletal/guideline-for-the-management-of-knee-and-hipoa-2nd-edition.pdf (2018).
22. McAlindon TE, LaValley MP, Harvey WF, et al. Effect of Intra-articular Triamcinolone vs Saline on Knee Cartilage Volume and Pain in Patients With Knee Osteoarthritis: A Randomized Clinical Trial. *Jama* 2017; 317: 1967-1975. 2017/05/17. DOI: 10.1001/jama.2017.5283.
23. Karim Z, Wakefield RJ, Quinn M, et al. Validation and reproducibility of ultrasonography in the detection of synovitis in the knee: a comparison with arthroscopy and clinical examination. *Arthritis and rheumatism* 2004; 50: 387-394. 2004/02/12. DOI: 10.1002/art.20054.
24. Naredo E, Cabero F, Beneyto P, et al. A randomized comparative study of short term response to blind injection versus sonographic-guided injection of local corticosteroids in patients with painful shoulder. *The Journal of rheumatology* 2004; 31: 308-314. 2004/02/05.
25. Altman R, Asch E, Bloch D, et al. Development of criteria for the classification and reporting of osteoarthritis. Classification of osteoarthritis of the knee. Diagnostic and Therapeutic Criteria Committee of the American Rheumatism Association. *Arthritis and rheumatism* 1986; 29: 1039-1049. 1986/08/01. DOI: 10.1002/art.1780290816.
26. Hunter DJ, Guermazi A, Lo GH, et al. Evolution of semi-quantitative whole joint assessment of knee OA: MOAKS (MRI Osteoarthritis Knee Score). *Osteoarthritis and cartilage* 2011; 19: 990-1002. 2011/06/08. DOI: 10.1016/j.joca.2011.05.004.
27. Han W, Aitken D, Zhu Z, et al. Signal intensity alteration in the infrapatellar fat pad at baseline for the prediction of knee symptoms and structure in older adults: a cohort study. *Annals of the rheumatic diseases* 2016; 75: 1783-1788. 2015/11/28. DOI: 10.1136/annrheumdis-2015-208360.
28. da Costa BR, Saadat P, Basciani R, et al. Visual Analogue Scale has higher assay sensitivity than WOMAC pain in detecting between-group differences in treatment effects: a meta-epidemiological study. *Osteoarthritis and cartilage* 2021; 29: 304-312. 2020/12/04. DOI: 10.1016/j.joca.2020.10.004.
29. Symonds T, Hughes B, Liao S, et al. Validation of the Chinese Western Ontario and McMaster Universities Osteoarthritis Index in Patients From Mainland China With Osteoarthritis of the Knee. *Arthritis care & research* 2015; 67: 1553-1560. 2015/05/29. DOI: 10.1002/acr.22631.

30. Hawthorne G, Richardson J and Osborne R. The Assessment of Quality of Life (AQoL) instrument: a psychometric measure of health-related quality of life. *Quality of life research : an international journal of quality of life aspects of treatment, care and rehabilitation* 1999; 8: 209-224. 1999/09/03. DOI: 10.1023/a:1008815005736.
31. Galenkamp H, Stronks K, Snijder MB, et al. Measurement invariance testing of the PHQ-9 in a multi-ethnic population in Europe: the HELIUS study. *BMC psychiatry* 2017; 17: 349. 2017/10/27. DOI: 10.1186/s12888-017-1506-9.
32. Cai G, Aitken D, Laslett LL, et al. Effect of Intravenous Zoledronic Acid on Tibiofemoral Cartilage Volume Among Patients With Knee Osteoarthritis With Bone Marrow Lesions: A Randomized Clinical Trial. *Jama* 2020; 323: 1456-1466. 2020/04/22. DOI: 10.1001/jama.2020.2938.
33. Ruan G, Yang C, Meng T, et al. Associations between diet quality and knee joint structures, symptoms and systemic abnormalities in people with symptomatic knee osteoarthritis. *Clinical nutrition (Edinburgh, Scotland)* 2021; 40: 2483-2490. 2021/05/02. DOI: 10.1016/j.clnu.2021.03.011.
34. Cai J, Xu J, Wang K, et al. Association Between Infrapatellar Fat Pad Volume and Knee Structural Changes in Patients with Knee Osteoarthritis. *The Journal of rheumatology* 2015; 42: 1878-1884. 2015/08/16. DOI: 10.3899/jrheum.150175.
35. Jin X, Jones G, Cicuttini F, et al. Effect of Vitamin D Supplementation on Tibial Cartilage Volume and Knee Pain Among Patients With Symptomatic Knee Osteoarthritis: A Randomized Clinical Trial. *Jama* 2016; 315: 1005-1013. 2016/03/10. DOI: 10.1001/jama.2016.1961.
36. Wang Z, Jones G, Winzenberg T, et al. Effectiveness of Curcuma longa Extract for the Treatment of Symptoms and Effusion-Synovitis of Knee Osteoarthritis : A Randomized Trial. *Annals of internal medicine* 2020; 173: 861-869. 2020/09/15. DOI: 10.7326/m20-0990.

GLucocorticoid injections into InfrapaTellar faT pad in patients with knee ostEoarthRitiS (GLITTERS)

STATISTICAL ANALYSIS PLAN (SAP)

Table of content

1. **Administrative information** 2
   1.1. **Title, registration, versions and revisions** 2
   1.2. **Signatures** 2
   1.3. **Abbreviations** 2
2. **Introduction** 3
   2.1. **Background and rationale** 3
   2.2. **Objectives** 3
3. **Study methods** 4
   3.1. **Trial design** 4
   3.2. **Randomization and blinding** 4
   3.3. **Sample size estimation** 4
   3.4. **Timing of final analysis** 5
   3.5. **Timing of outcome assessment** 5
4. **Statistical Principles** 6
   4.1. **Confidence Intervals and P-values** 6
   4.2. **Adherence and protocol deviations** 6
5. **Trial population** 6
   5.1. **Eligibility** 6
   5.2. **Withdraw/follow-up** 7
   5.3. **Baseline patient characteristics** 7
6. **Analysis** 8
   6.1. **Outcome definition** 8
   6.2. **Analysis methods** 8
   6.3. **Missing data** 9
   6.4. **Statistical software** 9

References 9

1. Administrative information

1.1. Title, registration, versions and revisions

Study Title	Efficacy and safety of glucocorticoid injections into infrapatellar fat pad in patients with knee osteoarthritis: a multicentre, double-blind, randomized clinical trial
Trial Registration Number	NCT05291650
Trial Statistician	Peichun Gao
Trial Principal Investigator	Changhai Ding
SAP Author(s)	Yan Zhang

1.2. Signatures

1.2.1. First Author

Name:

Yan Zhang

....

Date:

1.2.2. Statisticians

Name:

Peichun Gao

....

Date:

1.2.3. Principle investigator

Name:

....

Date:

1.3. Abbreviations

GLucocorticoid injections into InfrapaTellar faT pad in patients with knee osteoarthritis (GLITTERS), Statistical analysis plan (SAP), osteoarthritis (OA), infrapatellar fat pad (IPFP), Visual Analogue Scale (VAS), Western Ontario and McMaster Universities Osteoarthritis Index (WOMAC), Research Electronic Data Capture (REDCap), Assessment of Quality of Life-4 Dimensions (AQoL-4D), and Patient Health Questionnaire-9 (PHQ-9), adverse event (AE), magnetic resonance imaging (MRI), minimum clinically important difference (MCID).

2. Introduction

2.1. Background and rationale

Inflammatory phenotype is one of the most typical phenotypes of knee osteoarthritis (OA)[1-3]. Infrapatellar fat pad (IPFP) and synovium as a structural complex are considered as the main source of inflammation in knee OA. Glucocorticoid is a traditional anti-inflammatory drug for knee OA. Clinically, intra-articular glucocorticoid injections are recommended for people with knee OA to reduce their pain[4, 5]. However, emerging evidence has shown that repeated intra-articular injections over 2 years would induce more cartilage loss[6]. Thus, the injection of glucocorticoid into IPFP may not only play a better role in anti-inflammatory but minimize cartilage deterioration among inflammatory knee OA patients. To date, there is no study about the injection of drugs into IPFP. Therefore, we hypothesized that the IPFP injection of glucocorticoid can effectively reduce the knee pain and effusion-synovitis volume, compared to the placebo injection among people with symptomatic knee OA who have effusion- and Hoffa-synovitis.

2.2. Objectives

- Primary aim: to assess whether the injection of glucocorticoid into the IPFP can effectively reduce the knee pain measured by visual analogue scale (VAS) and MRI measured effusion-synovitis volume, compared to the placebo injection among patients with symptomatic knee OA who have both effusion- and Hoffa-synovitis.
- Secondary aims: to evaluate whether the injection of glucocorticoid into the IPFP effectively reduces the total Western Ontario and McMaster Universities Osteoarthritis Index (WOMAC) score, MRI-assessed Hoffa-synovitis score,

and pain medication use; improves the quality of life; and has a difference in IPFP volume and adverse reaction, compared to the placebo injection among symptomatic knee OA patients with both effusion- and Hoffa-synovitis.

3. Study methods

3.1. Trial design

GLucocorticoid injections into InfrapaTellar faT pad in patients with knee osteoarthritis (GLITTERS) is a multicentre, randomized, double-blind, placebo-controlled trial over 12 weeks. Participant recruitment is conducted across four centres: Zhujiang Hospital of Southern Medical University, the Sixth Affiliated Hospital of Sun Yat-sen University, the Third Affiliated Hospital of Sun Yat-sen University, and Beihai People's Hospital. This study will use competitive enrolment.

3.2. Randomization and blinding

Using block randomization in block size of four, eligible participants in each centre will be assigned to the treatment or placebo group with a 1:1 allocation rate based on computer-generated random numbers. A staff member who is not involved in this trial will pack the drugs with a random number indicated on each of the packages. Allocation concealment will be ensured and Allocation results will be not revealed until the completion of final data analyses. Double-blind (participants & researchers including outcome assessors and statisticians) design will be applied in this trial. Because of the different appearance of the injection drugs, the physical therapists for the injections are not blinded, and they will not be involved in other processes of this trial. During the period of preparing and injecting the drug, participants will be out of sight of the injection drugs. Unblinding will be conducted after all the data analyses are obtained. Emergency unblinding will be permissible when a serious adverse event (AE) happens, and patients who are unblinded will be withdrawn from the trial.

3.3. Sample size estimation

We estimated the sample size based on the primary outcomes. The formula $n_1=n_2=2\times[(Z_\alpha+Z_\beta)\times\sigma/\delta]^2$ was used to calculate the sample size with an α level of 0.05 (two-sided; $Z_\alpha=1.96$) and a power of 80% ($Z_\beta=0.842$). It is reported that the minimum clinically important difference (MCID) of knee VAS pain in people with knee OA is 19.9 mm. Assuming that the knee VAS pain reduction in the glucocorticoid injection

group compared with the saline injection group is clinically significant, the difference between knee VAS pain changes of the two groups should be at least 19.9 mm (δ). As the standard deviation of the change in knee VAS pain from baseline to three months follow-up is 24.2 mm (σ) in our previous trial[7], the $n_1=n_2$ calculated is 24. Considering a 20% loss to follow-up, a sample size of 30 patients in each group is needed.

As an MCID for effusion-synovitis volume has not yet been defined, the detectable difference of effusion–synovitis volume between the treatment and placebo groups were calculated based on the given sample size ($n_1=n_2=30$) and σ. According to the standard deviation of the change in effusion-synovitis volume from baseline to 12 weeks follow-up being 7.69 ml (σ) in the same previous trial[8], the detectable difference of effusion–synovitis volume calculated is 6.22 ml.

3.4. Timing of final analysis

After receiving SAP approval from Zhujiang Hospital's Clinical Research Centre and completing data collection, we will initiate data cleaning and validation. Subsequent to these procedures, the final analysis will be executed.

3.5. Timing of outcome assessment

Upon completion of the MRI follow-up assessments for all participants, the evaluation will proceed with MRI assessments. We will utilize sagittal images obtained from intermediate-weighted/proton density-weighted fat suppression sequences for the quantification and scoring of effusion-synovitis volume/score, Hoffa-synovitis score, tibiofemoral bone marrow lesion maximum size, tibiofemoral cartilage defects, and IPFP volume.

Data from questionnaires, including VAS, WOMAC, AQoL-4D, and PHQ-9, as well as information on medication and supplement use, AEs, and adverse reactions, will be systematically documented and managed via the Research Electronic Data Capture (REDCap) system at each visit. This tool is a secure, web-based software designed specifically for research data management, featuring: (1) a user-friendly interface for accurate data entry; (2) comprehensive audit trails for monitoring data alterations and exports; (3) automated export functions for efficient data transfer to popular statistical analysis software; and (4) capabilities for integrating data from external sources. Additionally, backups of de-identified data will be securely stored in password-protected electronic formats.

4. Statistical Principles

4.1. Confidence Intervals and *P*-values

A 2-sided *P* value of 0.050 will be treated as statistically significant. Given the lack of correlation between the two primary outcomes, which reflect distinct aspects of OA symptom and structural changes, we will refrain from adjusting the *P*-value for multiplicity in their analysis.

4.2. Adherence and protocol deviations

Major protocol deviations are defined as instances where participants are not eligible, have not been randomized and blinded, have not completed all four follow-ups, or exhibit significant deviations from the prescribed time windows, specifically exceeding four weeks.

5. Trial population

5.1. Eligibility

- Inclusion criteria:

1) Diagnosed with symptomatic knee OA according to American College of Rheumatology criteria[9];
2) Age > 45 years;
3) Have knee pain for more than six months and the pain assessed by VAS (100 mm) ≥ 40 mm in last week;
4) Ultrasonography showed obvious synovitis (over about 10 ml) with effusion in the knee joint;
5) Both MRI-assessed Hoffa-synovitis score (MRI Osteoarthritis Knee Score, MOAKS method)[10, 11] and effusion-synovitis score (MOAKS method)[10] ≥ 1, and their total score ≥ 3;
6) Being able to listen, speak, read and understand Chinese, capable of understanding the study requirements and cooperating with the researchers during the study, and providing written informed consent.

- Exclusion criteria

1) Allergy to glucocorticoids;
2) Knee joint injection of glucocorticoid or hyaluronic acid within the past six months;

3) Severe trauma or arthroscopy in the knee within the past six months;
4) Planned hip or knee surgery (including arthroscopy, arthroplasty, and other open joint surgeries) in the next six months;
5) Contraindication to having MRI (e.g., implanted pacemaker, artificial metal valve or cornea, aneurysm clipping surgery, arterial dissection, metal foreign bodies in the eyeball, claustrophobia);
6) Presence of other arthritis, such as rheumatoid arthritis and psoriatic arthritis;
7) Other condition that is more painful than their knee OA;
8) Malignant tumors or other life-threatening diseases;
9) Infection, diabetes, coagulopathy, osteonecrosis, or gastric/duodenal ulcer within the past 12 months;
10) Current use of oral corticosteroids, nonsteroidal anti-inflammatory drugs, or immunosuppressive medication;
11) Pregnancy or lactating female;
12) Use any investigational drugs or devices in the recent 30 days.

Note: When both knees of the participants meet the eligibility criteria, the knee with more severe VAS pain will be selected as the study knee.

5.2. Withdraw/follow-up

The participants are allowed to withdraw at any time throughout the study. If the participants withdraw from the study before the end of the trial, they are asked to have data collected including MRI assessment. The reason and date of the withdrawal will be recorded and the data before the withdrawal will be asked to retain.

In instances where participants experience a serious adverse event leading to their unblinding, such circumstances will be considered equivalent to withdrawal from the trial. Participants who cannot be reached or fail to return within a 16-week timeframe will be classified as lost to follow-up.

5.3. Baseline patient characteristics

Baseline factors such as demographic data (gender, birth date, height, weight, and study site), questionnaire responses (VAS, WOMAC, AQoL-4D, PHQ-9), and information on medication and supplement usage will be collected prior to the intervention. These will be analysed by randomized treatment arm to compare the groups. However, any differences in baseline characteristics will be attributed to chance rather than bias.

6. Analysis

6.1. Outcome definition

- Primary outcomes: changes in knee pain as assessed by the VAS and effusion-synovitis volume measured on MRI.
- Secondary outcomes: changes in the total score of WOMAC, MRI-detected Hoffa-synovitis score, quality of life measured using AQoL-4D, pain medication use, IPFP volume, and the incidence of adverse reactions.

6.2. Analysis methods

Baseline characteristics will be displayed in descriptive statistics according to the data type. Continuous variables with normal distribution will be displayed by means and standards deviation. Continuous variables with non-normal distribution will be displayed by median and interquartile range. Category variables will be displayed by proportion.

The intention-to-treat analysis will be the primary analyses method. The per-protocol analysis will be the second analyses method where the per-protocol population is defined as participants completed four follow-ups without major protocol deviations. There is no interim analysis in this research. Mixed-effects regression models will be used to calculate treatment group differences with continuous measures. In the models, fixed effects will be follow-up time, baseline covariates (including age, sex, BMI, baseline value of the corresponding outcome), treatment, and interactions between follow-up time and baseline covariates and treatment. Study site and individual participant identification will be treated as the random intercepts and follow-up time as the random slop in the models. The overall treatment group differences will be calculated by the linear combination of the estimated coefficients. Wilcoxon rank-sum test will be used to analyse the difference in pain medication use between the treatment and placebo groups. Pain medication use will be classified as commenced/increased, unchanged, or discontinued/decreased. The chi-square test or Fisher's exact test will be used to compare the incidence of adverse reactions between the two treatment groups, and the number of adverse reactions will be reported as the number of participants reporting at least one adverse reaction.

6.3. Missing data

In our study, we anticipate no missing data for baseline characteristics, including age, sex, BMI, and study site. For the outcomes, we assume that any missing values are missing at random. To address these missing values, we used multiple imputations with chained equations. Imputations were performed independently for each group, based on baseline variables (age, sex, BMI, and study site) and non-missing values at other time points. Additionally, multiple responses were treated as missing.

6.4. Statistical software

All analyses will be performed using SAS version 9.4 (SAS Institute, Institute, Carey, North Carolina, USA) or R4.0.3 (The R Foundation of Statistical Computing).

References

1. Deveza LA, Nelson AE and Loeser RF. Phenotypes of osteoarthritis: current state and future implications. *Clinical and experimental rheumatology* 2019; 37 Suppl 120: 64-72. 2019/10/18.

2. Dell'Isola A, Allan R, Smith SL, et al. Identification of clinical phenotypes in knee osteoarthritis: a systematic review of the literature. *BMC musculoskeletal disorders* 2016; 17: 425. 2016/10/14. DOI: 10.1186/s12891-016-1286-2.

3. Mobasheri A, Saarakkala S, Finnilä M, et al. Recent advances in understanding the phenotypes of osteoarthritis. *F1000Research* 2019; 8 2019/12/31. DOI: 10.12688/f1000research.20575.1.

4. Kolasinski SL, Neogi T, Hochberg MC, et al. 2019 American College of Rheumatology/Arthritis Foundation Guideline for the Management of Osteoarthritis of the Hand, Hip, and Knee. *Arthritis & rheumatology (Hoboken, NJ)* 2020; 72: 220-233. 2020/01/08. DOI: 10.1002/art.41142.

5. Practitioners RACoG. Guideline for the management of knee and hip osteoarthritis, https://www.racgp.org.au/download/Documents/Guidelines/Musculoskeletal/guideline-for-the-management-of-knee-and-hipoa-2nd-edition.pdf (2018).

6. McAlindon TE, LaValley MP, Harvey WF, et al. Effect of Intra-articular Triamcinolone vs Saline on Knee Cartilage Volume and Pain in Patients With Knee Osteoarthritis: A Randomized Clinical Trial. *Jama* 2017; 317: 1967-1975. 2017/05/17. DOI: 10.1001/jama.2017.5283.

7. Jin X, Jones G, Cicuttini F, et al. Effect of Vitamin D Supplementation on Tibial Cartilage Volume and Knee Pain Among Patients With Symptomatic Knee Osteoarthritis: A Randomized Clinical Trial. *Jama* 2016; 315: 1005-1013. 2016/03/10. DOI: 10.1001/jama.2016.1961.

8. Wang Z, Jones G, Winzenberg T, et al. Effectiveness of Curcuma longa Extract for the Treatment of Symptoms and Effusion-Synovitis of Knee Osteoarthritis : A Randomized Trial. *Ann Intern Med* 2020; 173: 861-869. 2020/09/15. DOI: 10.7326/m20-0990.

9. Altman R, Asch E, Bloch D, et al. Development of criteria for the classification and reporting of osteoarthritis. Classification of osteoarthritis of the knee. Diagnostic and

Therapeutic Criteria Committee of the American Rheumatism Association. *Arthritis and rheumatism* 1986; 29: 1039-1049. 1986/08/01. DOI: 10.1002/art.1780290816.
10. Hunter DJ, Guermazi A, Lo GH, et al. Evolution of semi-quantitative whole joint assessment of knee OA: MOAKS (MRI Osteoarthritis Knee Score). *Osteoarthritis and cartilage* 2011; 19: 990-1002. 2011/06/08. DOI: 10.1016/j.joca.2011.05.004.
11. Han W, Aitken D, Zhu Z, et al. Signal intensity alteration in the infrapatellar fat pad at baseline for the prediction of knee symptoms and structure in older adults: a cohort study. *Annals of the rheumatic diseases* 2016; 75: 1783-1788. 2015/11/28. DOI: 10.1136/annrheumdis-2015-208360.
