# Supplementary material for: Infrapatellar Fat Pad Glucocorticoid Injection in Knee Osteoarthritis: A Randomized Clinical Trial
Source: JAMA Netw Open. 2026 Jan 2;9(1):e2549938. doi: 10.1001/jamanetworkopen.2025.49938 (PMC12761338; doi:10.1001/jamanetworkopen.2025.49938)
Supplement: Supplement 2. — eMethods. eTable 1. Magnetic Resonance Imaging Sequences and Parameters Across the 4 Study Sites eTable 2. Intrareader and Interreader Reliability and Smallest Detectable Change for MRI Outcomes eTable 3. Sample Sizes of Participants With Complete Data for Each Group at Each Follow-Up Time Point eTable 4. Detailed Results for Intention-to-Treat Analyses Using Mixed-Effect Model eTable 5. Within- and Between-Participant Standard Deviations for Outcomes Using Mixed Model for Repeated Measures eTable 6. Per-Protocol Analysis of 12-Week Changes in All Outcomes Using Mixed-Effects Regression eTable 7. Detailed Results for Per-Protocol Analyses Using Mixed-Effect Model eTable 8. Pain Medication Use Details in Participants Reporting Increased Use eTable 9. Adverse Events eFigure. Intention-to-Treat Analysis of Changes in Post Hoc Outcomes With 4 Visits: WOMAC Pain, WOMAC Stiffness, WOMAC Function, and PHQ-9 [file jamanetwopen-e2549938-s002.pdf]

Supplemental Online Content

Zhang Y, Ruan G, Fan T, et al. Infrapatellar fat pad glucocorticoid injection in knee osteoarthritis: a randomized clinical trial. *JAMA Netw Open.* 2026;9(1):e2549938. doi:10.1001/jamanetworkopen.2025.49938

eMethods.

eTable 1. Magnetic Resonance Imaging Sequences and Parameters Across the 4 Study Sites

eTable 2. Intrareader and Interreader Reliability and Smallest Detectable Change for MRI Outcomes

eTable 3. Sample Sizes of Participants With Complete Data for Each Group at Each Follow-Up Time Point

eTable 4. Detailed Results for Intention-to-Treat Analyses Using Mixed-Effect Model

eTable 5. Within- and Between-Participant Standard Deviations for Outcomes Using Mixed Model for Repeated Measures

eTable 6. Per-Protocol Analysis of 12-Week Changes in All Outcomes Using Mixed-Effects Regression

eTable 7. Detailed Results for Per-Protocol Analyses Using Mixed-Effect Model

eTable 8. Pain Medication Use Details in Participants Reporting Increased Use

eTable 9. Adverse Events

eFigure. Intention-to-Treat Analysis of Changes in Post Hoc Outcomes With 4 Visits: WOMAC Pain, WOMAC Stiffness, WOMAC Function, and PHQ-9

This supplemental material has been provided by the authors to give readers additional information about their work.

© 2026 Zhang Y et al. *JAMA Network Open*.

eMethods

Assessment of secondary outcomes

Secondary outcomes included the Western Ontario and McMaster Universities Osteoarthritis Index (WOMAC), quality of life, infrapatellar fat pad (IPFP) volume, Hoffa-synovitis score, pain medication use, and adverse reactions. The WOMAC[1] evaluates patients' pain (five items), stiffness (two items), and functional dysfunction (17 items) over the last week, with each item measured on a 100-mm visual analog scale. The WOMAC score is calculated by summing the scores of each item, with a higher WOMAC score indicating more severe OA symptoms. Quality of life was assessed using the four-dimensional Assessment of Quality of Life (AQoL-4D)[2] questionnaire, which evaluates four dimensions including independent living, social relationships, psychological well-being, and physical senses over the last week. In this questionnaire, a higher score represents a higher quality of life. IPFP volume was measured by manually outlining the IPFP boundaries on section-by-section images using OsiriX software, with the volume calculated by the program[3]. The Hoffa-synovitis score was scored using MRI Osteoarthritis Knee Score (MOAKS)[4,5], based on discrete areas of increased signal intensity within the IPFP. Grades ranged from 0 to 3, where grade 0 = normal; grade 1 = signal increase < 10%; grade 2 = signal increase 10–20%; and grade 3 = signal increase > 20%. Pain medication use was determined by the researchers from the records of medication use and categorized as commenced/increased, unchanged, or discontinued/decreased. Details of the adverse event were recorded at each follow-up, with the researcher determining whether they qualified as adverse reactions.

Assessment of post-hoc outcomes

Post-hoc outcomes included the WOMAC pain, stiffness, and function subscale scores, depression, bone marrow lesion size, cartilage defects, and effusion-synovitis score. The WOMAC subscales[1] assess pain (five items), stiffness (two items), and functional limitation (17 items) over the past week, with each item measured on a 100-mm visual analog scale. Depression was evaluated using the nine-item Patient Health Questionnaire (PHQ-9)[6], which consists of nine items rated on a four-point scale (0–3), yielding a total score ranging from 0 to 27, with higher scores indicating more severe depressive symptoms. The bone marrow lesions size was assessed at the medial tibial, medial femoral, lateral tibial, and lateral femoral compartments. For each compartment, the slice showing the largest lesion area was identified by comparing adjacent slices[7]. The bone marrow lesions size was calculated as the sum of the maximum lesion sizes across the four compartments. Cartilage defects were graded using a modified Outerbridge classification: grade 0 = normal; grade 1 = focal blistering or intra-cartilaginous hyperintensity with a normal contour; grade 2 = surface irregularities and < 50% thickness loss; grade 3 = deep ulceration with > 50% thickness loss without subchondral bone exposure; and grade 4 = full-thickness loss with subchondral bone exposure[8]. Cartilage defects were assessed in the medial tibial, medial femoral, lateral tibial, and lateral femoral compartments, and the tibiofemoral cartilage defect score was calculated as the sum of scores across these four compartments. The effusion-synovitis score was assessed using the MOAKS and graded on a 0–3 scale: 0 = physiologic amount; 1 = small fluid collection continuous in the retropatellar space; 2 = medium, with slight convexity of the suprapatellar bursa; 3 = large, with evidence of capsular distention[4]. Higher scores indicate more severe effusion-synovitis.

Assessment of the smallest detectable change in the MRI assessments

For MRI assessments, including effusion-synovitis volume, IPFP volume, Hoffa-synovitis score, bone marrow lesion, cartilage defect, and effusion-synovitis score, the smallest detectable change (SDC) was calculated using the following formula[9]: $SDC = SEM_{CHANGE\text{-}SCORE} \times 1.96 \times \sqrt{2}$, where SEM refers to the standard error of measurement.

Handling of missing data

Missing data, including those with multiple answers treated as missing, were handled as follows. For WOMAC data, as specified in the protocol, if fewer than five items were incomplete, the total WOMAC score was calculated by averaging the available item scores and multiplying the result by 24.

© 2026 Zhang Y et al. *JAMA Network Open*.

When more than five items were not completed, the WOMAC score would be regarded as missing data. For missing data during follow-up, multiple imputation with chained equations was used if the assumption of missing at random was plausible[10]. The detailed steps for multiple imputation are as follows: the imputation procedure was initialized using the mean of the observed values from corresponding time points within each study group. Specifically, 20 imputations were independently generated based on baseline variables (age, sex, body mass index, and study site) and non-missing values from other time points, assuming the data were missing at random within each group. Each imputed dataset was then analyzed using a mixed model for repeated measures, and the resulting parameter estimates were combined following Rubin's rules to account for within- and between-imputation variability[11].

References

1. Symonds T, Hughes B, Liao S, Ang Q, Bellamy N. Validation of the Chinese Western Ontario and McMaster Universities Osteoarthritis Index in Patients From Mainland China With Osteoarthritis of the Knee. *Arthritis Care Res (Hoboken)*. Nov 2015;67(11):1553–60. doi:10.1002/acr.22631
2. Hawthorne G, Richardson J, Osborne R. The Assessment of Quality of Life (AQoL) instrument: a psychometric measure of health-related quality of life. *Quality of life research : an international journal of quality of life aspects of treatment, care and rehabilitation*. May 1999;8(3):209–24. doi:10.1023/a:1008815005736
3. Cai J, Xu J, Wang K, et al. Association Between Infrapatellar Fat Pad Volume and Knee Structural Changes in Patients with Knee Osteoarthritis. *J Rheumatol*. Oct 2015;42(10):1878–84. doi:10.3899/jrheum.150175
4. Hunter DJ, Guermazi A, Lo GH, et al. Evolution of semi-quantitative whole joint assessment of knee OA: MOAKS (MRI Osteoarthritis Knee Score). *Osteoarthritis Cartilage*. Aug 2011;19(8):990–1002. doi:10.1016/j.joca.2011.05.004
5. Han W, Aitken D, Zhu Z, et al. Signal intensity alteration in the infrapatellar fat pad at baseline for the prediction of knee symptoms and structure in older adults: a cohort study. *Ann Rheum Dis*. Oct 2016;75(10):1783–8. doi:10.1136/annrheumdis-2015-208360
6. Galenkamp H, Stronks K, Snijder MB, Derks EM. Measurement invariance testing of the PHQ-9 in a multi-ethnic population in Europe: the HELIUS study. *BMC psychiatry*. Oct 24 2017;17(1):349. doi:10.1186/s12888-017-1506-9
7. Cai G, Aitken D, Laslett LL, et al. Effect of Intravenous Zoledronic Acid on Tibiofemoral Cartilage Volume Among Patients With Knee Osteoarthritis With Bone Marrow Lesions: A Randomized Clinical Trial. *Jama*. Apr 21 2020;323(15):1456–1466. doi:10.1001/jama.2020.2938
8. Ruan G, Yang C, Meng T, et al. Associations between diet quality and knee joint structures, symptoms and systemic abnormalities in people with symptomatic knee osteoarthritis. *Clin Nutr*. May 2021;40(5):2483–2490. doi:10.1016/j.clnu.2021.03.011
9. Dontje ML, Dall PM, Skelton DA, Gill JMR, Chastin SFM. Reliability, minimal detectable change and responsiveness to change: Indicators to select the best method to measure sedentary behaviour in older adults in different study designs. *PloS one*. 2018;13(4):e0195424. doi:10.1371/journal.pone.0195424
10. Jakobsen JC, Gluud C, Wetterslev J, Winkel P. When and how should multiple imputation be used for handling missing data in randomised clinical trials - a practical guide with flowcharts. *BMC medical research methodology*. Dec 6 2017;17(1):162. doi:10.1186/s12874-017-0442-1
11. Rubin DB. *Multiple Imputation for Nonresponse in Surveys*. Wiley; 2004.

© 2026 Zhang Y et al. *JAMA Network Open*.

eTable 1. Magnetic Resonance Imaging Sequences and Parameters Across the 4 Study Sites

Study site	Machine and coil	Proton density-weighted sagittal
Zhujiang Hospital of Southern Medical University	3.0 T whole-body MR unit (Philips Medical Systems; Philips-DF3S9QI), using a dedicated 8-channel receive knee coil	PDW TSE SPAIR (Proton Density Weighted Turbo Spin-Echo Spectral Attenuated Inversion Recovery) Sag acquisition;<br>flip angle 90 degrees;<br>repetition time 4230.9 ms;<br>echo time 30 ms;<br>field of view 94 mm;<br>864 × 864 matrix;<br>slice thickness 2 mm
The Sixth Affiliated Hospital of Sun Yat-sen University	1.5 T whole-body MR unit (UIH; uMR 570), using a dedicated transmit receive knee coil (VTC)	3D PD FSE FS (Proton Density with Fast Spin-Echo Fat Suppression) Sag acquisition;<br>flip angle 130 degrees;<br>repetition time 4842 ms;<br>echo time 35 ms;<br>field of view 100 mm;<br>340 × 272 matrix;<br>slice thickness 2 mm
The Third Affiliated Hospital of Sun Yat-sen University	3.0 T whole-body MR unit (GE Medical Systems; DISCOVERY MR750), using a dedicated receive knee coil (HD TR Knee PA)	3D FS PD (Fat-Suppressed Proton-Density) Sag acquisition;<br>flip angle 142 degrees;<br>repetition time 3839 ms;<br>echo time 31.3 ms;<br>field of view 100 mm;<br>512 × 512 matrix;<br>slice thickness 2 mm
Beihai People's Hospital	3.0 T whole-body MR unit (SIEMENS Medical Systems; AWP145994), using a dedicated 15-channel transmit receive knee coil	3D PD TSE FS (Proton Density with Turbo Spin-Echo Fat Suppression) Sag acquisition;<br>flip angle 180 degrees;<br>repetition time 3000 ms;<br>echo time 31 ms;<br>field of view 100 mm;<br>320 × 320 matrix;<br>slice thickness 2 mm

© 2026 Zhang Y et al. *JAMA Network Open*.

eTable 2. Intrareader and Interreader Reliability and Smallest Detectable Change for MRI Outcomes

Outcomes in MRI	Intra-reader reliability	Inter-reader reliability*	Smallest detectable change#
Effusion-synovitis volume (ml)	0.99	0.75	2.36
Infrapatellar fat pad volume (ml)	0.96	0.92	0.26
Hoffa-synovitis score	0.75	0.65	0.17
Bone marrow lesion (cm^2)	0.94	0.87	0.45
Cartilage defect	0.97	0.84	0.40
Effusion-synovitis score	0.88	0.79	0.19

MRI: magnetic resonance imaging.

* Two observers independently measured the outcomes.

# Calculated using the following formula: Smallest detectable change = $SEM_{CHANGE\text{-}SCORE} \times 1.96 \times \sqrt{2}$, where SEM refers to the standard error of measurement.

© 2026 Zhang Y et al. *JAMA Network Open*.

eTable 3. Sample Sizes of Participants With Complete Data for Each Group at Each Follow-Up Time Point

Outcomes	Group	Baseline	Week 4	Week 8	Week 12
Primary outcomes					
VAS	Treatment	30	30	30	30
	Placebo	30	30	30	30
Effusion-synovitis volume	Treatment	30	-	-	30
	Placebo	30	-	-	30
Secondary outcomes					
WOMAC total	Treatment	30	30	30	30
	Placebo	30	28[a]	30	30
AQoL-4D	Treatment	29[b]	30	29[c]	28[a]
	Placebo	30	29[d]	29[c]	29[c]
Infrapatellar fat pad volume	Treatment	30	-	-	30
	Placebo	30	-	-	30
Hoffa-synovitis score	Treatment	30	-	-	30
	Placebo	30	-	-	30
Pain medication use	Treatment	30	30	30	30
	Placebo	30	30	30	30
Adverse reaction	Treatment	-	30	30	30
	Placebo	-	30	30	30
Post hoc outcomes					
WOMAC pain	Treatment	30	30	30	30
	Placebo	30	30	30	30
WOMAC stiffness	Treatment	30	30	30	30
	Placebo	30	30	30	30
WOMAC function	Treatment	30	30	30	30
	Placebo	30	28[a]	30	30
PHQ-9	Treatment	30	30	29[c]	29[c]
	Placebo	30	30	30	30
Bone marrow lesion	Treatment	30	-	-	30
	Placebo	30	-	-	30
Cartilage defect	Treatment	30	-	-	30
	Placebo	30	-	-	30
Effusion-synovitis score	Treatment	30	-	-	30
	Placebo	30	-	-	30

VAS: visual analog scale; WOMAC: Western Ontario and McMaster University Index; AQoL-4D: four-dimensional Assessment of Quality of Life; PHQ-9: nine-items Patient Health Questionnaire.

a: both two participants have one missing item;

b: one participant has two missing items. The score was calculated by averaging the remaining items and then multiplying by 12;

c: one participant has one missing item;

d: one participant has three missing items.

© 2026 Zhang Y et al. *JAMA Network Open*.

eTable 4. Detailed Results for Intention-to-Treat Analyses Using Mixed-Effect Model

Outcome	Follow-up time point		Treatment (N = 30)	Placebo (N = 30)	Between group difference in least squares mean change (95% CI)	*P* value	Standardized mean difference (95% CI)
VAS	Baseline	Mean (SD)	66.0 (16.6)	64.0 (18.4)			
	Week 4	Mean (SD)	29.5 (25.6)	38.8 (26.4)			
		Change from baseline (95% CI)	-37.6 (-49.4, -25.9)	-27.2 (-38.9, -15.5)	-10.4 (-22.2, 1.4)	0.084	-0.37 (-0.88, 0.14)
	Week 8	Mean (SD)	29.3 (24.82)	34.0 (28.9)			
		Change from baseline (95% CI)	-36.5 (-48.3, -24.7)	-30.5 (-42.2, -18.8)	-6.0 (-17.8, 5.8)	0.32	-0.21 (-0.72, 0.29)
	Week 12	Mean (SD)	27.3 (25.16)	33.0 (29.60)			
		Change from baseline (95% CI)	-39.3 (-51.1, -27.5)	-31.4 (-43.1, -19.7)	-7.9 (-19.7, 4.0)	0.19	-0.28 (-0.79, 0.23)
WOMAC total	Baseline	Mean (SD)	911.5 (484.0)	972.5 (439.8)			
	Week 4	Mean (SD)	547.0 (572.6)	743.9 (612.5)			
		Change from baseline (95% CI)	-363.7 (-535.8, -191.7)	-224.7 (-392.9, -56.6)	-139.0 (-378.9, 100.9)	0.25	-0.30 (-0.81, 0.21)
	Week 8	Mean (SD)	568.8 (571.9)	697.3 (674.5)			
		Change from baseline (95% CI)	-343.4 (-522.7, -164.2)	-252.4 (-427.6, -77.2)	-91.0 (-341.0, 159.0)	0.47	-0.19 (-0.70, 0.32)
	Week 12	Mean (SD)	424.3 (500.3)	616.0 (646.1)			
		Change from baseline (95% CI)	-479.6 (-636.3, -322.8)	-345.2 (-498.4, -192.0)	-134.4 (-353.0, 84.2)	0.22	-0.32 (-0.83, 0.19)
AQoL-4D	Baseline	Mean (SD)	81.3 (12.8)	83.9 (10.1)			
	Week 4	Mean (SD)	86.3 (10.8)	84.9 (12.5)			
		Change from baseline (95% CI)	4.6 (1.7, 7.6)	1.4 (-1.5, 4.3)	3.2 (-0.9, 7.3)	0.13	0.39 (-0.12, 0.90)
	Week 8	Mean (SD)	87.9 (9.7)	86.3 (12.3)			
		Change from baseline (95% CI)	6.1 (3.7, 8.6)	2.6 (0.2, 4.9)	3.6 (0.2, 7.0)	0.039	0.53 (0.02, 1.05)
	Week 12	Mean (SD)	88.6 (9.3)	87.5 (11.9)			
		Change from baseline (95% CI)	7.0 (4.3, 9.7)	3.4 (0.8, 6.1)	3.6 (-0.2, 7.4)	0.062	0.48 (-0.03, 0.99)
WOMAC pain	Baseline	Mean (SD)	189.4 (104.4)	200.0 (109.6)			
	Week 4	Mean (SD)	102.3 (114.0)	138.9 (127.8)			
		Change from baseline (95% CI)	-89.5 (-125.6, -53.3)	-56.4 (-91.7, -21.1)	-33.1 (-83.3, 17.2)	0.19	-0.34 (-0.85, 0.17)
	Week 8	Mean (SD)	98.8 (114.21)	140.8 (138.8)			
		Change from baseline (95% CI)	-95.6 (-132.9, -58.4)	-53.9 (-90.3, -17.6)	-41.7 (-93.4, 10.1)	0.11	-0.41 (-0.93, 010)
	Week 12	Mean (SD)	79.5 (97.4)	127.5 (129.75)			
		Change from baseline (95% CI)	-113.0 (-144.5, -81.4)	-66.8 (-97.6, -36.0)	-46.2 (-90.0, -2.4)	0.039	-0.54 (-1.06, -0.03)
WOMAC stiffness	Baseline	Mean (SD)	53.7 (57.2)	71.3 (54.0)			
	Week 4	Mean (SD)	39.9 (47.5)	56.8 (52.2)			
		Change from baseline (95% CI)	-19.5 (-35.7, -3.3)	-8.6 (-24.4, 7.1)	-10.9 (-33.6, 11.9)	0.34	-0.25 (-0.76, 0.26)
	Week 8	Mean (SD)	40.9 (47.49)	47.9 (56.15)			

© 2026 Zhang Y et al. *JAMA Network Open*.

		Change from baseline (95% CI)	-20.3 (-37.8, -2.8)	-16.6 (-33.7, 0.4)	-3.6 (-28.2, 20.9)	0.77	-0.08 (-0.58, 0.43)
	Week 12	Mean (SD)	29.0 (43.3)	41.0 (52.5)			
		Change from baseline (95% CI)	-29.8 (-45.2, -14.3)	-24.7 (-39.7, -9.6)	-5.1 (-26.8, 16.6)	0.64	-0.12 (-0.63, 0.38)
WOMAC function	Baseline	Mean (SD)	668.4 (350.7)	701.2 (310.3)			
	Week 4	Mean (SD)	404.8 (420.8)	537.9 (452.2)			
		Change from baseline (95% CI)	-260.7 (-387.2, -134.3)	-154.9 (-278.6, -31.2)	-105.8 (-282.1, 70.5)	0.23	-0.31 (-0.82, 0.20)
	Week 8	Mean (SD)	429.1 (423.8)	508.6 (486.9)			
		Change from baseline (95% CI)	-233.7 (-364.7, -102.7)	-176.9 (-305.0, -48.8)	-56.8 (-239.4, 125.8)	0.54	-0.16 (-0.67, 0.35)
	Week 12	Mean (SD)	315.8 (364.1)	447.4 (473.0)			
		Change from baseline (95% CI)	-343.9 (-461.0, -226.9)	-248.1 (-362.6, -133.6)	-95.8 (-259.0, 67.4)	0.24	-0.30 (-0.81, 0.21)
PHQ-9	Baseline	Mean (SD)	4.4 (4.4)	4.9 (4.7)			
	Week 4	Mean (SD)	3.1 (3.9)	4.2 (4.4)			
		Change from baseline (95% CI)	-1.9 (-3.7, -0.0)	-1.3 (-3.1, 0.6)	-0.6 (-2.3, 1.0)	0.46	-0.12 (-0.62, 0.39)
	Week 8	Mean (SD)	2.6 (3.4)	4.3 (4.1)			
		Change from baseline (95% CI)	-2.6 (-4.4, -0.7)	-1.3 (-3.1, 0.6)	-1.3 (-3.0, 0.4)	0.12	-0.25 (-0.76, 0.26)
	Week 12	Mean (SD)	2.6 (3.2)	3.8 (3.69)			
		Change from baseline (95% CI)	-2.6 (-4.5, -0.7)	-1.8 (-3.7, 0.1)	-0.8 (-2.5, 0.9)	0.35	-0.15 (-0.66, 0.35)

VAS: visual analog Scale; WOMAC: Western Ontario and McMaster University Index; AQoL-4D: four-dimensional Assessment of Quality of Life; PHQ-9: nine-items Patient Health Questionnaire.

© 2026 Zhang Y et al. *JAMA Network Open*.

eTable 5. Within- and Between-Participant Standard Deviations for Outcomes Using Mixed Model for Repeated Measures

Outcomes	Within-subject standard deviation	Between-subject standard deviation
VAS	12.4	20.5
WOMAC total	228.3	395.1
AQoL-4D	4.6	5.7
WOMAC pain	43.2	86.4
WOMAC stiffness	23.5	37.8
WOMAC function	174.7	287.3
PHQ-9	2.1	2.4

VAS: visual analog Scale; WOMAC: Western Ontario and McMaster University Index; AQoL-4D: four-dimensional Assessment of Quality of Life; PHQ-9: nine-items Patient Health Questionnaire.

© 2026 Zhang Y et al. *JAMA Network Open*.

eTable 6. Per-Protocol Analysis of 12-Week Changes in All Outcomes Using Mixed-Effects Regression

Outcomes	Treatment* (N = 29)	Placebo* (N = 29)	Between group difference in least squares mean change (95% CI)	*P* value
Primary outcomes				
VAS, mm	-40.6 (-53.7, -27.4)	-34.4 (-47.5, -21.3)	-6.2 (-18.0, 5.6)	0.30
Effusion-synovitis volume, ml	-4.3 (-6.8, -1.7)	-5.3 (-7.8, -2.8)	1.0 (-1.2, 3.3)	0.37
Secondary outcomes				
WOMAC total	-491.0 (-649.6, -332.4)	-370.0 (-526.3, -213.7)	-121.0 (-342.7, 100.7)	0.28
AQoL-4D	7.1 (4.4, 9.9)	4.0 (1.2, 6.7)	3.2 (-0.7, 7.0)	0.11
Infrapatellar fat pad volume, ml	0.5 (0.2, 0.8)	0.3 (0.0, 0.6)	0.2 (-0.2, 0.6)	0.35
Hoffa-synovitis score	-0.2 (-0.5, 0.2)	-0.1 (-0.4, 0.2)	-0.0 (-0.3, 0.2)	0.72
Change of pain medication use†				
Commenced/increased, n (%)	3 (10.3)	9 (31.0)	-	0.052
Reporting at least one adverse reaction				
Yes, n (%)	1 (3.4)	1 (3.4)	-	0.99
Post-hoc outcomes				
WOMAC pain	-114.9 (-145.8, -84.0)	-74.7 (-105.2, -44.3)	-40.2 (-83.3, 2.9)	0.067
WOMAC stiffness	-30.8 (-46.3, -15.3)	-28.8 (-44.1, -13.5)	-2.0 (-23.9, 19.9)	0.86
WOMAC function	-351.6 (-471.3, -231.8)	-261.4 (-379.4, -143.4)	-90.2 (-257.4, 77.0)	0.28
PHQ-9	-2.6 (-4.5, -0.7)	-1.8 (-3.8, 0.1)	-0.7 (-2.5, 1.0)	0.40
Bone marrow lesion (cm^2)	-0.4 (-1.1, 0.3)	-0.3 (-1.0, 0.4)	-0.1 (-0.7, 0.5)	0.82
Cartilage defect	-0.0 (-1.0, 0.9)	0.5 (-0.5, 1.4)	-0.5 (-1.0, 0.0)	0.056
Effusion-synovitis score	-0.2 (-0.4, -0.0)	-0.2 (-0.4, -0.0)	-0.0 (-0.3, 0.3)	0.97

VAS: visual analog scale; WOMAC: Western Ontario and McMaster University Index; AQoL-4D: four-dimensional Assessment of Quality of Life; PHQ-9: nine-items Patient Health Questionnaire; CI: confidence interval.

* For continuous outcomes, values represented the least squares mean change from baseline to 12 weeks (95% CI). Between-group differences in least squares mean change with 95% CI were derived from mixed-effects models, adjusted for follow-up time, baseline covariates (age, sex, body mass index, baseline value of the corresponding outcome), treatment, and interactions between follow-up time and baseline covariates, as well as treatment. For categorical outcomes (including change of pain medication and reporting at least one adverse reaction), values were presented as actual counts with percentages. The chi-squared test or Fisher's exact test was used.

† Pain medication use was categorized as "commenced/increased," "unchanged," or " discontinued/decreased." No participants in either group had discontinued or decreased their medication.

© 2026 Zhang Y et al. *JAMA Network Open*.

eTable 7. Detailed Results for Per-Protocol Analyses Using Mixed-Effect Model

Outcome	Follow-up time point		Treatment (N = 30)	Placebo (N = 30)	Between group difference in mean change (95% CI)	*P* value
VAS	Baseline	Mean (SD)	66.4 (16.7)	63.3 (18.4)		
	Week 4	Mean (SD)	28.7 (25.8)	37.0 (25.0)		
		Change from baseline (95% CI)	-40.6 (-53.7, -27.4)	-29.9 (-43.1, -16.8)	-10.6 (-22.4, 1.2)	0.077
	Week 8	Mean (SD)	28.6 (25.01)	32.2 (27.6)		
		Change from baseline (95% CI)	-39.5 (-52.6, -26.4)	-32.9 (-46.0, -19.8)	-6.6 (-18.4, 5.2)	0.27
	Week 12	Mean (SD)	28.2 (25.1)	31.2 (28.3)		
		Change from baseline (95% CI)	-40.6 (-53.7, -27.4)	-34.4 (-47.5, -21.3)	-6.2 (-18.0, 5.6)	0.30
WOMAC total	Baseline	Mean (SD)	930.3 (481.3)	955.4 (437.3)		
	Week 4	Mean (SD)	526.2 (571.0)	709.6 (593.3)		
		Change from baseline (95% CI)	-406.6 (-574.9, -238.4)	-239.0 (-404.8, -73.2)	-167.6 (-402.8, 67.5)	0.16
	Week 8	Mean (SD)	549.2 (571.7)	657.1 (648.8)		
		Change from baseline (95% CI)	-388.9 (-562.8, -215.0)	-269.5 (-440.9, -98.1)	-119.4 (-362.5, 123.6)	0.33
	Week 12	Mean (SD)	437.7 (503.7)	575.9 (618.5)		
		Change from baseline (95% CI)	-491.0 (-649.6, -332.4)	-370.0 (-526.3, -213.7)	-121.0 (-342.7, 100.7)	0.28
AQoL-4D	Baseline	Mean (SD)	81.3 (12.8)	83.9 (10.1)		
	Week 4	Mean (SD)	86.3 (10.8)	84.9 (12.5)		
		Change from baseline (95% CI)	5.0 (2.0, 7.9)	1.9 (-1.0, 4.9)	3.1 (-1.1, 7.2)	0.15
	Week 8	Mean (SD)	87.9 (9.7)	86.3 (12.3)		
		Change from baseline (95% CI)	6.5 (4.1, 9.0)	3.1 (0.7, 5.5)	3.4 (0.0, 6.8)	0.048
	Week 12	Mean (SD)	88.6 (9.3)	87.5 (11.9)		
		Change from baseline (95% CI)	7.1 (4.4, 9.9)	4.0 (1.2, 6.7)	3.2 (-0.7, 7.0)	0.11
WOMAC pain	Baseline	Mean (SD)	192.1 (105.2)	199.0 (111.4)		
	Week 4	Mean (SD)	103.1 (115.9)	132.5 (125.0)		
		Change from baseline (95% CI)	-92.7 (-129.5, -56.0)	-61.9 (-98.1, -25.7)	-30.8 (-82.0, 20.4)	0.23
	Week 8	Mean (SD)	96.2 (115.3)	134.1 (136.2)		
		Change from baseline (95% CI)	-102.7 (-139.8, -65.7)	-58.8 (-95.3, -22.3)	-43.9 (-95.6, 7.8)	0.094
	Week 12	Mean (SD)	82.3 (97.9)	119.2 (123.6)		
		Change from baseline (95% CI)	-114.9 (-145.8, -84.0)	-74.7 (-105.2, -44.3)	-40.2 (-83.3, 2.9)	0.067
WOMAC stiffness	Baseline	Mean (SD)	55.2 (57.7)	72.6 (54.5)		
	Week 4	Mean (SD)	36.8 (45.2)	53.8 (50.5)		
		Change from baseline (95% CI)	-24.9 (-40.0, -9.9)	-12.5 (-27.4, 2.3)	-12.4 (-33.6, 8.9)	0.25

© 2026 Zhang Y et al. *JAMA Network Open*.

	Week 8	Mean (SD)	38.4 (46.3)	44.5 (53.9)		
		Change from baseline (95% CI)	-25.2 (-41.6, -8.7)	-21.2 (-37.3, -5.0)	-4.0 (-27.2, 19.2)	0.73
	Week 12	Mean (SD)	30.0 (43.7)	38.6 (51.6)		
		Change from baseline (95% CI)	-30.8 (-46.3, -15.3)	-28.8 (-44.1, -13.5)	-2.0 (-23.9, 19.9)	0.86
WOMAC function	Baseline	Mean (SD)	683.0 (347.5)	683.9 (300.8)		
	Week 4	Mean (SD)	386.3 (415.7)	523.4 (429.0)		
		Change from baseline (95% CI)	-294.5 (-417.1, -171.9)	-159.9 (-280.8, -39.1)	-134.6 (-305.8, 36.6)	0.12
	Week 8	Mean (SD)	414.6 (423.7)	478.6 (466.3)		
		Change from baseline (95% CI)	-266.8 (-394.3, -139.2)	-184.8 (-310.5, -59.0)	-82.0 (-260.1, 96.2)	0.36
	Week 12	Mean (SD)	325.4 (366.6)	418.1 (452.8)		
		Change from baseline (95% CI)	-351.6 (-471.3, -231.8)	-261.4 (-379.4, -143.4)	-90.2 (-257.4, 77.0)	0.28

VAS: visual analog Scale; WOMAC: Western Ontario and McMaster University Index; AQoL-4D: four-dimensional Assessment of Quality of Life; PHQ-9: nine-items Patient Health Questionnaire.

© 2026 Zhang Y et al. *JAMA Network Open*.

eTable 8. Pain Medication Use Details in Participants Reporting Increased Use

Medication	Administration route	Dose	Duration of use	Time of report	Study group
Imrecoxib	Oral	Two tablets daily	8 days	Week 8	Placebo
Ibuprofen(1); Celecoxib(2)	Oral(1); Oral(2)	0.3 g, six times daily(1); 0.2 g, once daily(2)	2 days(1); 3 days(2)	Week 4(1); Week 4(2)	Placebo
Diclofenac sodium	Oral	0.1 g, once daily	1 day	Week 4	Placebo
Diclofenac sodium	Oral	One tablet daily	Several days	Week 4	Placebo
Ibuprofen	Oral	One tablet twice daily	3 days	Week 8	Placebo
Ibuprofen	Oral	30 mg, twice daily	1 day	Week 12	Placebo
Aceclofenac(1); Celecoxib(2)	Oral(1); Oral(2)	0.1 g, twice daily(1); 0.2 g, twice daily(2)	Several days(1); Several days(2)	Week 4(1); Week 4(2)	Placebo
Diclofenac sodium	Oral	One tablet daily	1 day	Week 4	Placebo
Ibuprofen	Oral	0.3 g, three time daily	3 days	Week 4	Placebo
Diclofenac diethylamine emulgel	Topical	Apply a small amount	1 day	Week 4	Treatment
Nimesulide	Oral	0.1 g, twice daily	5 days	Week 12	Treatment
Diclofenac sodium	Oral	One tablet daily	1 day	Week 4	Treatment

Information on pain medication use was obtained through self-report during follow-up visits. For some participants, only the number of tablets was recorded because the exact dosage could not be recalled. Some participants increased the use of two medications, which are denoted as (1) and (2), respectively.

© 2026 Zhang Y et al. *JAMA Network Open*.

eTable 9. Adverse Events

Event	No. (%) of participants	
	Treatment (n = 30)	Placebo (n = 30)
Fatigue	0	1
Increased study knee pain the following day	1	1
Acute pharyngitis	0	1
Pain	1	1
Fever	0	1
Blood shortage in the brain	0	1
Falls	1	0
Cough	0	1

© 2026 Zhang Y et al. *JAMA Network Open*.

eFigure. Intention-to-Treat Analysis of Changes in Post Hoc Outcomes With 4 Visits: WOMAC Pain, WOMAC Stiffness, WOMAC Function, and PHQ-9

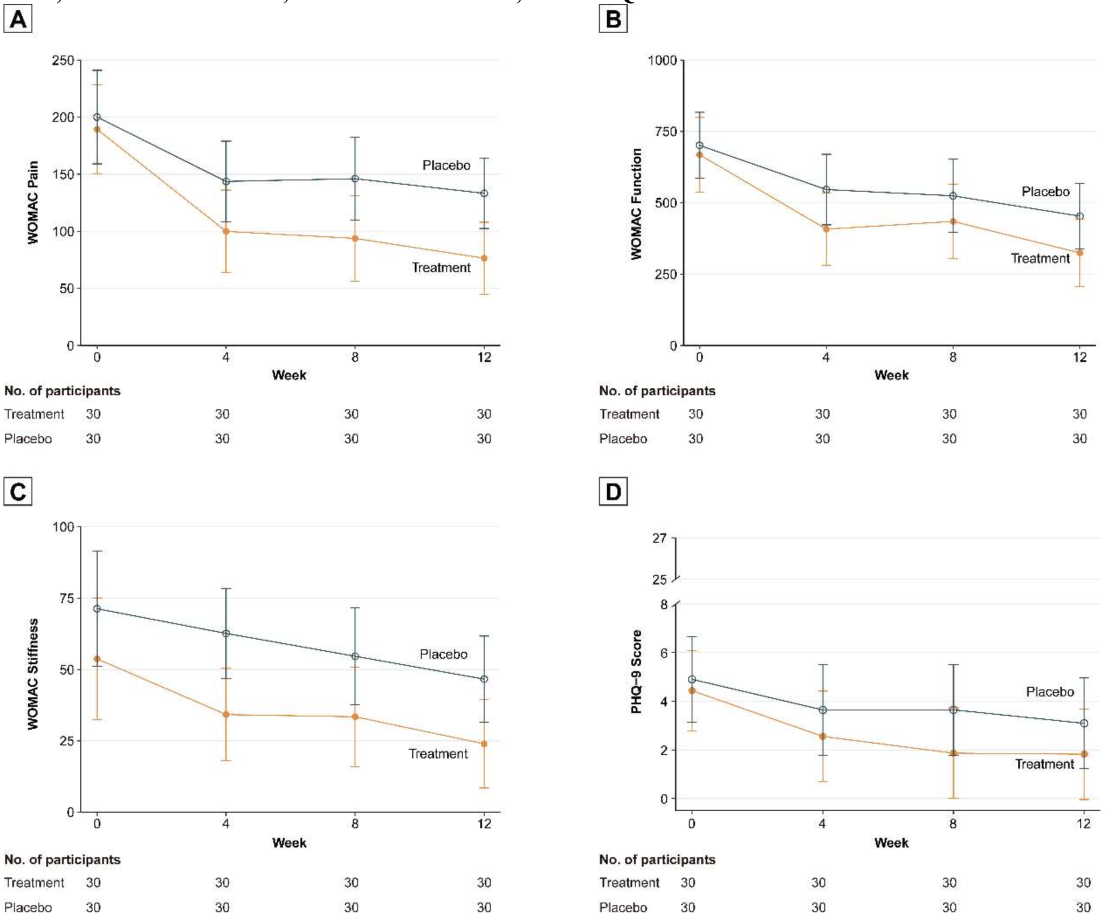

Abbreviations: WOMAC, Western Ontario and McMaster University Index; PHQ-9, nine-items Patient Health Questionnaire.

© 2026 Zhang Y et al. *JAMA Network Open*.
