# Supplementary material for: Infrapatellar Fat Pad Glucocorticoid Injection in Knee Osteoarthritis: A Randomized Clinical Trial
Source: JAMA Netw Open. 2026 Jan 2;9(1):e2549938. doi: 10.1001/jamanetworkopen.2025.49938 (PMC12761338; doi:10.1001/jamanetworkopen.2025.49938)
Supplement: Supplement 3. — Data Sharing Statement [file jamanetwopen-e2549938-s003.pdf]

Data Sharing Statement

Zhang. Infrapatellar Fat Pad Glucocorticoid Injection in Knee Osteoarthritis. *JAMA Netw Open*. Published January 02, 2026. doi:10.1001/jamanetworkopen.2025.49938

Data

Additional Information: ClinicalTrials.gov registration: NCT05291650. https://clinicaltrials.gov/study/NCT05291650?cond=NCT05291650.&rank=1
Data available: Yes
Data types: Data dictionary, Other (please specify)
Additional Information: All de-identified patient-level data are subject to restricted access and are not for commercial use. For academic access, please contact the corresponding author, Professor Changhai Ding (Changhai.Ding@utas.edu.au), with a detailed research request. Data will be provided once the request is approved. The sample code used for this study is publicly available on Github: https://github.com/yzhang666666/RCT-GLITTERS_2025.
How to access data: For academic access, please contact the corresponding author, Professor Changhai Ding (Changhai.Ding@utas.edu.au), with a detailed research request.
When available: With publication

Supporting Documents

Document types: Statistical/analytic code
How to access documents: The sample code used for this study is publicly available on Github: https://github.com/yzhang666666/RCT-GLITTERS_2025.
When available: With publication

Additional Information

Who can access the data: To researchers whose proposed use of the data has been approved.
Types of analyses: For academic purpose if approved by the corresponding author, Professor Changhai Ding (Changhai.Ding@utas.edu.au).
Mechanisms of data availability: The data will be made available after approval of a proposal.
